# Antifungal Efficacy against *Candida* spp. Strains and Synthesis via Heck–Matsuda Arylation of Aryl-Camphene-Type Derivatives

**DOI:** 10.1021/acsomega.5c11226

**Published:** 2026-03-20

**Authors:** Naiza Saraiva Farias, Laryssa de Souza-Salgado, Francisco Bernardo de Barros, Maria Alicy Neres de Oliveira, Márcia Jordana Ferreira Macedo, Francildo dos Santos Souza, João Pedro Alves Torres, Henrique Douglas Melo Coutinho, Jailton De Souza-Ferrari, Maria Audilene de Freitas, Maria Flaviana Bezerra Morais-Braga

**Affiliations:** † Department of Biological Chemistry, 226206 Regional University of Cariri, R. Cel. Antonio Luis 1161, Crato, Ceará 63105-000, Brazil; ‡ Department of Chemistry, 28097 Federal University of Paraiba, Campus I, João Pessoa, Paraíba 58051-900, Brazil

## Abstract

The rapid growth of fungal infections caused by *Candida* spp. associated with antimicrobial resistance requires the development of new therapeutic approaches to combat these infections. Camphene is a compound derived from the essential oils of various plants and has antimicrobial properties. Based on this, the objective of this study was to carry out the late-stage diversification of camphene by means of highly stereoselective Heck–Matsuda (HM) arylation and to evaluate the antifungal activity of these derivatives against *Candida* spp. The reaction provided direct access to seven aryl-camphene derivatives (adducts HM **3a**-**g**) with good to excellent yields (48–98%) in short reaction times under moderate and aerobic conditions. For the antifungal tests, camphene and the HM adducts **3a** and **3b**, with the highest yields, were selected. Of the compounds, camphene showed the best intrinsic antifungal activity, outperforming fluconazole (FCZ) against*C. albicans*. In association with FCZ, camphene and HM adduct **3a** significantly potentiated the drug’s action, resulting in a notable reduction in the average inhibitory concentration (IC_5_0). Tests to determine the minimum fungicidal concentration showed that only the combination of camphene and FCZ showed fungicidal action against*C. albicans*. For*C. tropicalis*, there was fungicidal activity with the combinations of camphene and FCZ, and HM adduct **3a** and FCZ. The morphological transition from yeast to hyphae and/or pseudohyphae in*C. albicans* was inhibited only by camphene. In*C. tropicalis*, filamentation was completely inhibited by the action of camphene and its derivatives at the concentrations evaluated. Taken together, these results suggest that camphene and its HM adducts (**3a**–**b**) evaluated may be promising candidates for the development of antifungal agents, especially in combination therapies with fluconazole.

## Graphical abstract

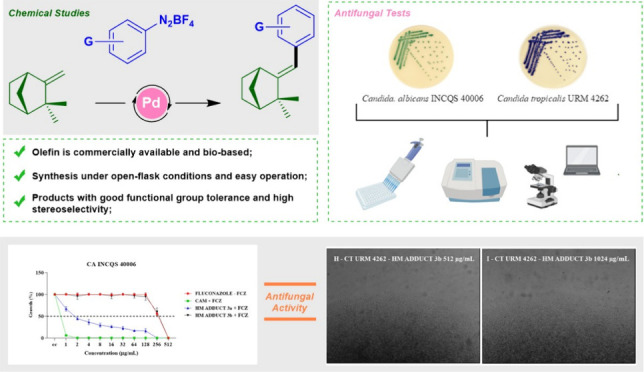

## 1 Introduction

*Candida* spp. are commensal and opportunistic fungi present in the human microbiota, primarily colonizing the skin, gastrointestinal tract, and reproductive tracts. Under normal conditions in the human body, these species do not cause any harm to the host; however, they can become opportunistic pathogens when there are alterations in the host’s microbiota or immune system, leading to the development of infections that range from superficial to systemic.^1,2^

The capacity to evade the host’s immune response is a crucial factor in the pathogenicity of *Candida* spp. Changes in morphology, expression of adhesins, biofilm formation, and secretion of hydrolytic enzymes are among the main factors that promote colonization, adhesion, invasion, and damage to the human body.^3–5^ These mechanisms associated with the significant increase in individuals with compromised immune systems have led to a vertiginous growth in fungal infections caused by *Candida* spp.^6,7^

Despite the high impact these infections have on human health and well-being, therapeutic options are limited to five classes of drugs: azoles, pyrimidine analogues, echinocandins, flucytosine, and polyenes.^8,9^ The azole class is commonly preferred for the treatment of *Candida* infections, with emphasis on fluconazole, a drug widely used by the healthcare system due to its low cost and low toxicity.^10,11^ However, *Candida* species have developed resistance to these classes of drugs, with resistance to azoles being more prevalent due to their widespread use and fungistatic nature.^12,13^

Thus, the problem of increased infections associated with fungal resistance constitutes a serious problem for the health system and requires urgent development of new bioactive molecules with antifungal potential.^14^ Therefore, investigations of natural products or biobased synthetic products have become good alternatives for the development of new antifungal drugs, whether intrinsically or in combination with other drugs.^15,16^

Camphene belongs to the terpene class and originates from the essential oils of plants, such as rosemary (*Rosmarinus officinalis*), valerian (*Valeriana officinalis*), and ginger (*Zingiber officinale*).^17–19^ Biological investigations have demonstrated that this compound and its derivatives possess pronounced antimicrobial, analgesic, and antitumor properties.^20–23^ Notably, the camphene-thiosemicarbazone-type derivatives are effective against*Mycobacterium tuberculosis* strains.^24,25^

Despite the immense biological potential of camphene for the development of novel antimicrobial prototypes, only a single study has explored the late-stage structural derivatization of this natural product.^26^ Given our interest in exploring Heck–Matsuda (HM) arylation as a strategy for late-stage structural diversification of biorelevant natural products,^27^ we decided to revisit the synthesis of biobased aryl-camphene-type derivatives through this approach to understand its scope and stereoselectivity.

Therefore, considering the scenario of increasing infections caused by *Candida* spp., the increase in fungal resistance, the need to develop new antifungal agents, and the biological properties of camphene, the objective of this study was to explore the late-stage diversification of camphene via HM arylation and evaluate the in vitro antifungal activity of camphene and its derivatives obtained in larger quantities, isolated and combined with fluconazole, against *Candida* spp. strains.

## 2 Materials and Methods

### 2.1 Chemical Studies

#### 2.1.1 Experimental Section

All experimental details, general methods (chromatography and instrumentation) employed in the synthetic studies of the late-stage structural diversification of camphene, and the tabulation of analytical and spectral data for each compound can be found in the Supporting Information (S.I.) file.

#### 2.1.2 Chemical Synthesis

General procedure for the Heck–Matsuda arylation of camphene (**1**). A typical method for the synthesis of aryl-camphene-type derivatives is as follows: A 20 mL test tube under an air atmosphere (open-flask) equipped with a magnetic stir bar (4.4 × 9.8 mm) was charged, in this order, with a solution of camphene (**1**; 68.12 mg; 0.5 mmol; 1.0 equiv) in 3 mL of absolute ethanol, ZnCO_3_ (94 mg; 0.75 mmol; 1.5 equiv), and Pd­(OAc)_2_ (11.2 mg; 0.05 mmol; 0.1 equiv.; 10 mol %). The mixture was stirred for approximately 5 min at 40 °C. After that, the corresponding arenediazonium salt (**2a**–**g**; 0,75 mmol; 1.5 equiv) was added in one portion, and the reaction mixture was stirred at 40 °C until TLC analysis indicated complete consumption of the olefin. After 1 h, the mixture was filtered through a small pad of silica gel (3.0 × 3.0 cm) eluted with ethyl acetate (4 × 10 mL) and concentrated in vacuo. The crude products were purified by flash column chromatography (on silica gel; 3.0 × 12 cm) with hexane/Et_2_O (98:2, v/v) as the eluent to give HM adducts **3a**–**b**; or just with hexane as the eluent to give HM adducts **3c**–**g**. Complete spectral data for each compound can be found in the S.I. file. The analytical and spectroscopic data for aryl-camphene-type derivatives **3b**–**e** are similar to those previously reported.^26^ The physical and spectroscopic data of **3f**–**g** are being described here for the first time.

### 2.2 In Vitro Antifungal Tests

#### 2.2.1 Compounds, Culture Media, and Reagents

For the antifungal tests, in addition to camphene and its derivatives, HM adduct **3a** and HM adduct **3b** were selected due to the quantitative availability of material. The antifungal drug fluconazole was used as a control. It was acquired from VIRUPAKSHA ORGANIC LIMITED (Gaddapotharam Village - India).

For the antifungal tests, double-concentrated Sabouraud Dextrose Agar (SDA, Himedia, Mumbai, India) and Sabouraud Dextrose Broth (SDB, KASVI, Condalab SA, Madrid, Spain) were used, prepared according to the manufacturers’ instructions. Potato Dextrose Agar (PDA, KASVI, Condalab SA, Madrid, Spain), Bacteriological Agar (BA, ISOFAR, Rio de Janeiro, Brazil), and Yeast Extract Peptone Dextrose (YPD, Difco, Becton, Dickinson and Company, Franklin Lakes, NJ, USA) were used to analyze the morphological transition. All the media were solubilized with distilled water and sterilized in a vertical autoclave at 121 °C for 15 min. Sterile Bovine Fetal Serum (LABORCLIN, Paraná, Brazil) and Tween 80 PS (DINÂMICA, São Paulo, Brazil) were used to analyze the morphological transition.

The compounds (camphene and its derivatives **3a**–**b**) and fluconazole were weighed in 0.01 g, dissolved in 1 mL of dimethyl sulfoxide (DMSO, VETEC Química Fina LTDA, Rio de Janeiro, Brazil), and then diluted in SDB medium to make up the initial concentration of 1024 μg/mL.

#### 2.2.2 Microorganisms

Two fungal strains were used: the standard strain *Candida albicans* INCQS 40006 (CA INCQS 40006) – ATCC 10231, obtained from the Culture Collection of the National Institute for Quality Control in Health (INCQS) of the Oswaldo Cruz Foundation (Rio de Janeiro, Brazil), and the isolated strain *Candida tropicalis* URM 4262 (CT URM 4262) from the Culture Collection of the Recife Mycotheque (URM – University Recife Mycology) of the Federal University of Pernambuco – UFPE.

#### 2.2.3 Inoculum Preparation

The strains were cultured in Petri dishes containing SDA at 35 °C for 24 h. The microorganisms were then added to test tubes containing 4 mL of 0.9% sodium chloride (NaCl) solution. They were adjusted and compared with the McFarland scale turbidity standard at 0.5 (1 × 10^8^ CFU/mL).^28^ For the fungal virulence inhibition tests, the yeasts were reactivated in YPD medium plus 5% fetal bovine serum and then cultivated in SDA at 37 °C for 24 h.

#### 2.2.4 Fungal Growth Curve and Determination of Inhibitory Concentration of 50% (IC_50_)

The fungal growth curve and the 50% Inhibitory Concentration (IC_50_) were obtained using the broth microdilution method in 96-well plates. In each well, 100 μL of the substances or fluconazole at concentrations ranging from 1 to 512 μg/mL, along with 100 μL of SDB containing fungal inoculum (28, with modifications to the concentrations), were added. The last two wells were used to check for fungal growth (11th) and sterility (12th). In addition, product dilution control plates were made using a 0.9% sodium chloride solution to replace the fungal inoculum. All the plates were incubated at 35 °C for 24 h, and then read in an ELISA spectrophotometer (Termoplate, Kasuaki, China) at a wavelength of 630 nm.^29^

#### 2.2.5 Determination of the Minimum Fungicidal Concentration (MFC)

The MFC was determined to evaluate the intrinsic and combined effects of the compounds and fluconazole on the cell viability of the *Candida* strains. Plates containing SDA were divided into quadrants by a guide card attached to the bottom outside of the plate. 10 μL of the solution from the microdilution plate prepared for this test was transferred to each quadrant. The plates were then incubated at 35 °C for 24 h. After this period, a visual reading was conducted to check for the presence or absence of *Candida* colony growth. MFC was defined as the lowest concentration capable of inhibiting the growth of fungal colonies.^30^

#### 2.2.6 Evaluation of the Combined Activity of the Compounds with Fluconazole

To evaluate the modifying effect of fluconazole action, the method proposed by Siqueira-Júnior and co-workers^31^ was used with some modifications. The compounds were tested based on the subinhibitory Matrix Concentration (MC/8). The plates were filled with 100 μL of a solution containing culture medium, compound, and inoculum, along with 100 μL of fluconazole diluted in concentrations ranging from 1 to 512 μg/mL. Growth control, sterility, and dilution of the compounds with fluconazole were carried out. All the plates were incubated at 35 °C for 24 h and then read in a spectrophotometric device at a wavelength of 630 nm (ELISA, Termoplate, Kasuaki, China).^29^

#### 2.2.7 Effect of Compounds and Fluconazole on the Morphological Transition of *Candida*

This test was conducted to verify the ability of the compounds and fluconazole to inhibit the transition from yeast to hyphae in *Candida* strains. Humid chambers containing sterile microscope slides were prepared. Each slide received 3 mL of solution containing PDA culture medium depleted with BA, Tween 80 PS, and Matrix Concentration – MC (1024 μg/mL) and MC/2 (512 μg/mL) of the compounds, along with fluconazole. After solidification, fungal subcultures were made with two parallel striations, then covered with sterile coverslips and incubated at 37 °C for 24 h. Growth controls were carried out on slides containing only PDA, which was depleted with BA and Tween. After the incubation period, the slides were viewed under an optical microscope L-2000I-TRINO/6633 (BIOVAL, São Paulo, Brazil) at 4× and 10× objectives, and images were captured. The total length of the striations and filaments expressed in the images was analyzed using the ToupView software (ToupTek Photonics—China).^32^

### 2.3 Statistical Analysis

Statistical analysis was conducted using *GraphPad Prism* 9.0 software. Data were expressed as geometric means, and statistical significance was verified by two-way ANOVA followed by Bonferroni *post hoc* test, where *p* < 0.05 and *p* < 0.0001 are considered significant, and *p* > 5 is not significant. IC_50_ values were obtained by nonlinear regression.

Five random images were selected from each concentration to measure the total length of the striations and hyphal or pseudohyphal structures. The average length of the filaments was calculated and analyzed by one-way ANOVA, followed by the Bonferroni post hoc test. The values were verified according to the concentration of the product.^32^

## 3 Results

### 3.1 Chemical Synthesis

Camphene (**1**) is an abundant monoterpene that is commercially available in both enantiomerically pure and racemic forms. However, the higher commercial cost of its optically active versions limits its use in synthetic optimization studies and biological screening efforts for early stage drug discovery. Furthermore, although the HM arylation of **1** has already been investigated in the past,^26^ the moderate yields obtained and the reaction times of some hours left room for further synthetic optimization. Therefore, in this work, we revisited the synthetic scope of the HM arylation of (±)-camphene (**1**), intending to access aryl-camphene derivatives potentially relevant for antifungal prospecting at low operational cost and high synthetic efficiency.

Synthetic studies began with the Heck–Matsuda reaction between (±)-camphene (**1**) and 4-methoxybenzenediazonium tetrafluoroborate (**2a**) as model coupling partners (Table 1). Initially, motivated by the tenability of a previously reported protocol,^26^ Zhuangyu′s catalytic system using Pd­(OAc)_2_ (0.9 mol %) was verified (Table S1, entry 1; see S.I. for details). However, in our hands, we could not replicate the functionality of this catalytic system. Changing the organic-aqueous catalytic system to an all-organic one, using only EtOH as a solvent, also had no effect (Table S1, entry 2; S.I.). On the other hand, the study of the impact of alcohol polarity on the performance of the reaction medium, the use of room-temperature conditions, and the evaluation of 1.0–1.5 equiv of some inorganic bases and 5–10 mol % of Pd­(OAc)_2_ made it possible to synthesize HM adduct (**3a**) in low-to-moderate yields in just 1 h (Table S1, entries 3–9; see S.I. for details). The isomer (*E*)-**3a** was detected as the only product by ^1^H NMR spectroscopy. Based on these studies, EtOH was established as the solvent of choice, and the optimal stoichiometric ratio of olefin to diazonium salt was verified as 1.0:1.5, using Pd­(OAc)_2_ (10 mol %) as the precatalyst (Table S1, entries 7–8). Further screening of 1.5 equiv well-known inorganic bases (Table S1, entries 10–13; see S.I. for details) showed the best synthetic efficiency under heating conditions at 40 °C for the preparation of **3a** in 95% yield, using ZnCO_3_ as the base (Table S1, entry 13). Attempts to fine-tune the HM arylation conditions, such as lowering the load of Pd­(OAc)_2_ from 10 to 5 mol % and decreasing the amount of diazonium salt from 1.5 to 1.2 equiv, provoked significant deterioration of yield (Table S1, entries 14–15; see S.I. for details).

**Table 1:**
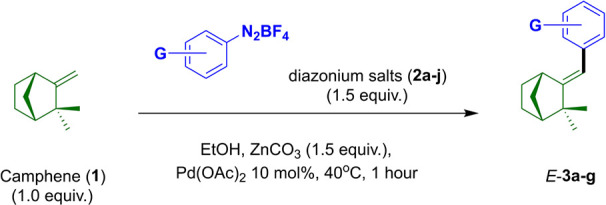

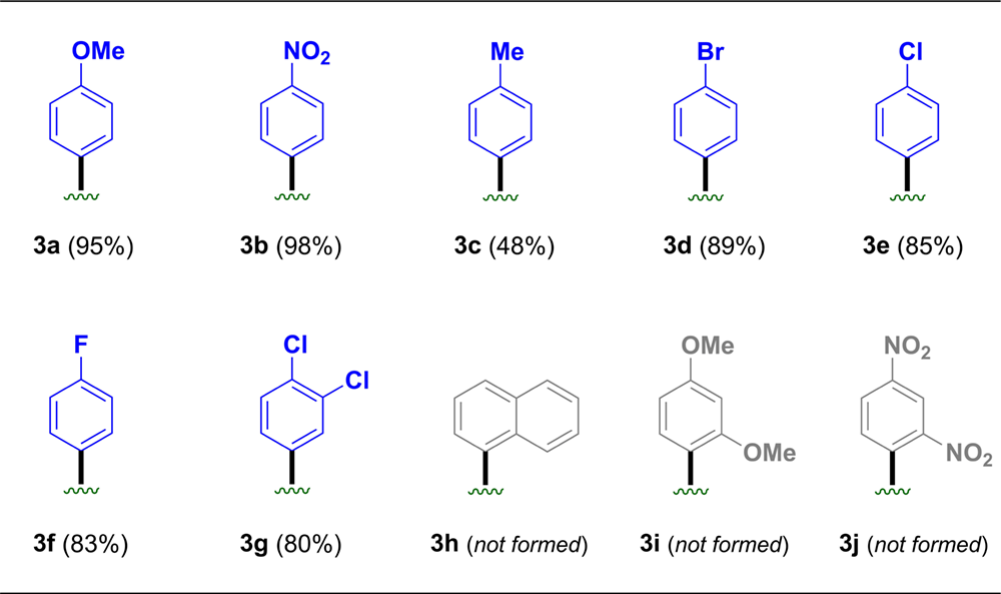
Scope of Late-Stage Diversification of Camphene (**1**) via Heck–Matsuda Arylation

With suitable conditions established, the scope of this Heck–Matsuda arylation process was evaluated (Table 1). Initially, the focus was on assessing the electronic effect of the substituents in various diazonium salts. The reactions of (±)-camphene (**1**) with both electron-rich and electron-deficient arenediazonium salts (**2**) furnished the corresponding aryl-camphene-type derivatives (HM adducts **3a**–**g**) as a single geometric isomer, identified as *E*, with yields ranging from moderate to excellent (up to 98%). Irrespective of steric influences, a low chemical yield was verified just for the *p*-methylbenzenediazonium salt **2c** in the synthesis of HM adduct **3c** (48%; Table 1). However, reducing the Pd­(OAc)_2_ load from 10 to 5 mol % made it possible to obtain this HM adduct also with a moderate yield (62%; Table S1, entry 20; see S.I. for details). Complete chemoselectivity was attained in the presence of halogen-substituted diazonium salts (**3d**–**3g**; Table 1). For example, 4-bromo-phenyl-camphene derivative **3d** was obtained in 89% yield and with high stereoselectivity, making this HM adduct a valuable substrate for further cross-coupling reactions. Unlike the electron effect, the steric effect of the substituents in the arenediazonium salts had a dramatic impact on the HM arylation process. The arylation of **1** with the *ortho*-substituted arenediazonium salts (**2h**–**j**) failed to afford the expected HM adducts, and the olefin remained unreacted in all cases (**3h**–**j**; Table 1). Furthermore, the yields were generally higher for the *para*-substituted salts than for the *meta*-substituted ones, which suggests a clear dependence between the synthetic efficiency and the steric effect. Finally, similar to what was observed for **3a**, only the *E*-isomers were observed for the other HM adducts, indicating that HM arylation was highly stereoselective in favor of the *E*-geometry.

### 3.2 Antifungal Tests

The results obtained from the analysis of the fungal growth curve and 50% inhibitory concentration (IC_50_) are shown in Figure 1 and Table 2. Figure 1A shows the fungal growth curve of CA INCQS 40006 and CT URM 4262 as a function of the tested concentrations of each compound intrinsically. For CA INCQS 40006, fluconazole (FCZ), the control drug, inhibited fungal cell growth by 100% at a concentration of 512 μg/mL. Camphene (CAM) showed an inhibition percentage of 98% (256 μg/mL), the HM adduct **3a** derivative inhibited fungal cell growth by 86.5% (512 μg/mL), and HM adduct **3b** inhibited it by 82% (512 μg/mL). For CT URM 4262, FCZ showed 100% inhibition at a concentration of 512 μg/mL. At the same concentration, CAM showed 99% inhibition of fungal growth, HM adduct **3a** showed 86%, and HM adduct **3b** showed 74%.

**Figure 1:**
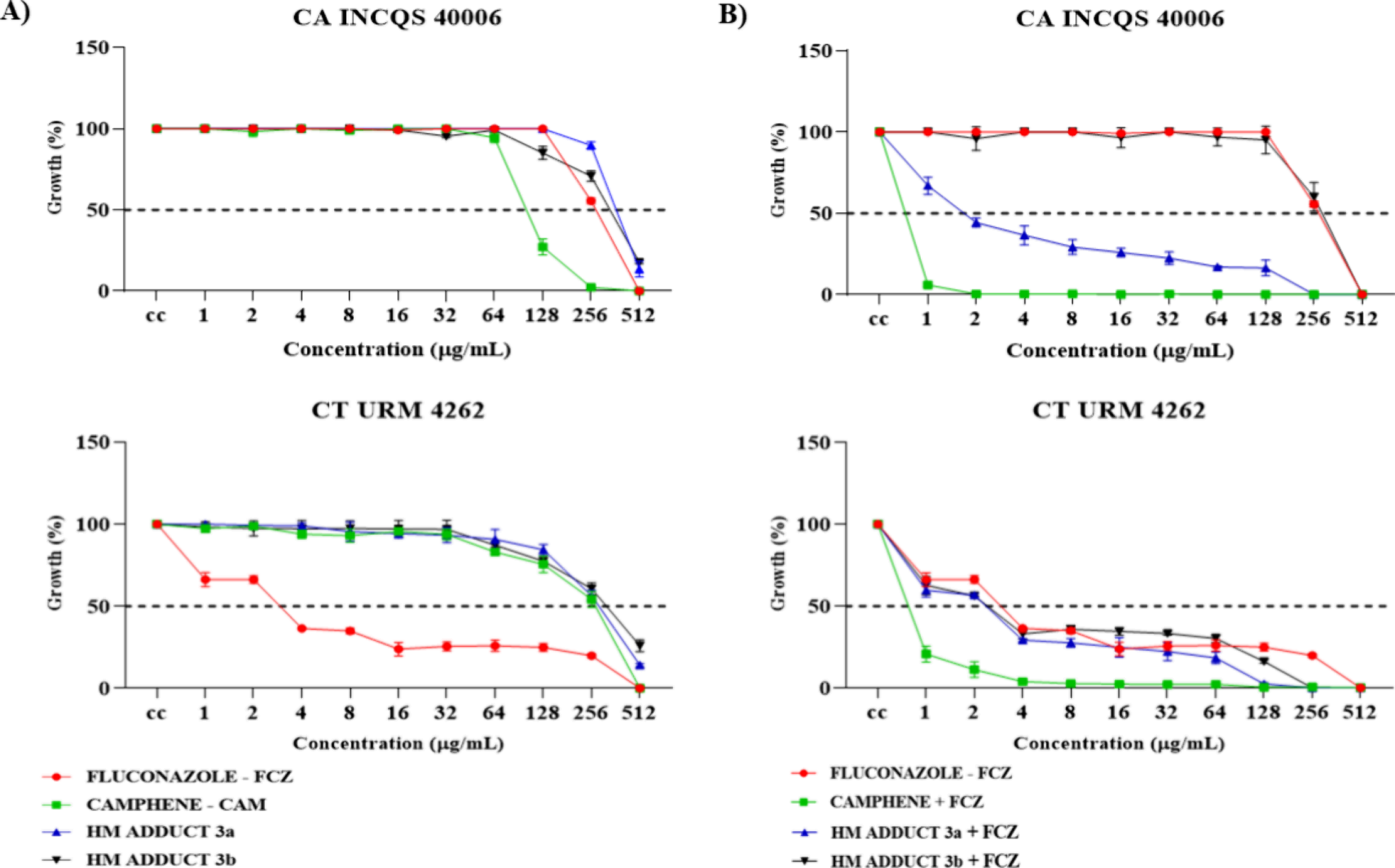
Fungal growth curve of *C. albicans* and *C. tropicalis* exposed to camphene, derivatives, and fluconazole intrinsically and in combination. (A) Intrinsic activity against *C. albicans* and *C. tropicalis*; (B) Combined activity against *C. albicans* and *C. tropicalis*. CA: *Candida albicans*; INCQS: National Institute for Health Quality Control, CT: *Candida tropicalis*; URM: University Recife Mycology; CC: Fungal growth control; FCZ: Fluconazole; CAM: Camphene; and HM: Heck Matsuda.

**Table 2:** 50% Inhibitory Concentration (IC_50_) of Camphene, Derivatives, Fluconazole (FCZ), and Their Combinations against *Candida albicans* and *Candida tropicalis* (μg/mL)^a^

	CA INCQS 40006	CT URM 4262
FCZ	256.7	3.177
CAMPHENE	106.9	222.3
HM adduct **3a**	371.8	268.0
HM adduct **3b**	323.0	294.3
CAM + FCZ	0.4901	0.2832
HM adduct **3a** + FCZ	2.021	1.962
HM adduct **3b** + FCZ	272.7	2.670

a CA: *Candida albicans*; CT: *Candida tropicalis*; INCQS: National Institute for Quality Control in Health; URM: – University Recife Mycology; FCZ: Fluconazole; CAM: Camphene (**1**); HM: Heck Matsuda; CAM + FCZ: Camphene combined with Fluconazole; HM adduct **3a** + FCZ**:** HM adduct **3a** combined with Fluconazole; HM adduct **3b** + FCZ: HM adduct **3b** combined with Fluconazole.

Figure 1B shows the fungal growth curve of CA INCQS 40006 and CT URM 4262 against the combined action of the compounds with FCZ. In the combined activity against CA INCQS 40006, the CAM + FCZ combination enhanced the drug’s inhibitory effect, achieving 100% inhibition of fungal growth at a concentration of 16 μg/mL. The combination HM adduct **3a** + FCZ also potentiated the action of the drug, with 100% inhibition of fungal growth at a concentration of 256 μg/mL. HM adduct **3b** + FCZ did not show significant results, obtaining 100% inhibition of fungal growth at the same concentration of the drug (512 μg/mL). Against CT URM 4262, the combination of CAM and FCZ inhibited fungal cell growth by 96% at a concentration of 4 μg/mL. HM adduct **3a** + FCZ showed 100% inhibition (256 μg/mL), a result similar to that obtained against CA INCQS 40006, and the combination HM adduct **3b** + FCZ showed 99% inhibition (256 μg/mL).

Table 2 presents the IC_50_ values obtained through the fungal growth curve. In intrinsic activity, against CA INCQS 40006, FCZ exhibited an IC_50_ at a concentration of 256.7 μg/mL, and CAM obtained an IC_50_ twice lower than FCZ at 106.9 μg/mL. HM adduct **3a** obtained a higher IC_50_ than FCZ, with 371.8 μg/mL, as did HM adduct **3b**, with an IC_50_ of 323.0 μg/mL. For CT URM 4262, FCZ exhibited an IC_50_ at a concentration of 3.177 μg/mL, while CAM obtained an IC_50_ approximately 70 times higher at 222.3 μg/mL. The HM adduct **3a** exhibited an IC_50_ higher than that of FCZ at 268.0 μg/mL, as did HM adduct **3b** with an IC_50_ of 294.3 μg/mL.

In the combined activity against CA INCQS 40006, CAM associated with FCZ reduced the IC_50_ of this drug from 256.7 to 0.4901 μg/mL, and the combination HM adduct **3a** + FCZ reduced it to 2.021 μg/mL. The combination of HM adduct **3b** + FCZ increased the IC_50_ from 256.7 to 272.7 μg/mL. Against CT URM 4262, CAM associated with FCZ reduced the IC_50_ from 3.177 to 0.2832 μg/mL. HM adduct **3a** + FCZ was able to reduce it to 1.962 μg/mL, and HM adduct **3b** + FCZ to 2.670 μg/mL.

In the CFM assays, it was not possible to observe a fungicidal effect from the intrinsic action of either the compounds or FCZ; however, in the combinations CAM + FCZ against CA INCQS 40006 and CT URM 4262 and HM adduct **3a** + FCZ against CT URM 4262, the CFM was observed at a concentration of 512 μg/mL.

For the analyses of the morphological transition, the change from yeast form to filamentous form (expression of hyphae and/or pseudohyphae) was used as a parameter. The statistical results are in Figure 2A,B. The growth of hyphae is expressed in the growth control of both strains. FCZ inhibited the development of these structures in CA INCQS 40006 and CT URM 4262 by 100% in all the concentrations evaluated. Figure 2A shows the results obtained against CA INCQS 40006; CAM inhibited filament growth by 100% in CM, but in CM/2, the result was not significant. HM adduct **3a** reduced the morphological transition of CA INCQS 40006 in CM by around 17%. In contrast, there was a light stimulus to filament growth in CM/2. HM adduct **3b** did not show significant results in inhibiting and reducing the filamentous structures of CA INCQS 40006. Figure 2B shows the results obtained against CT URM 4262, where the compounds completely inhibited the expression of pleomorphism in both CM and CM/2.

**Figure 2:**
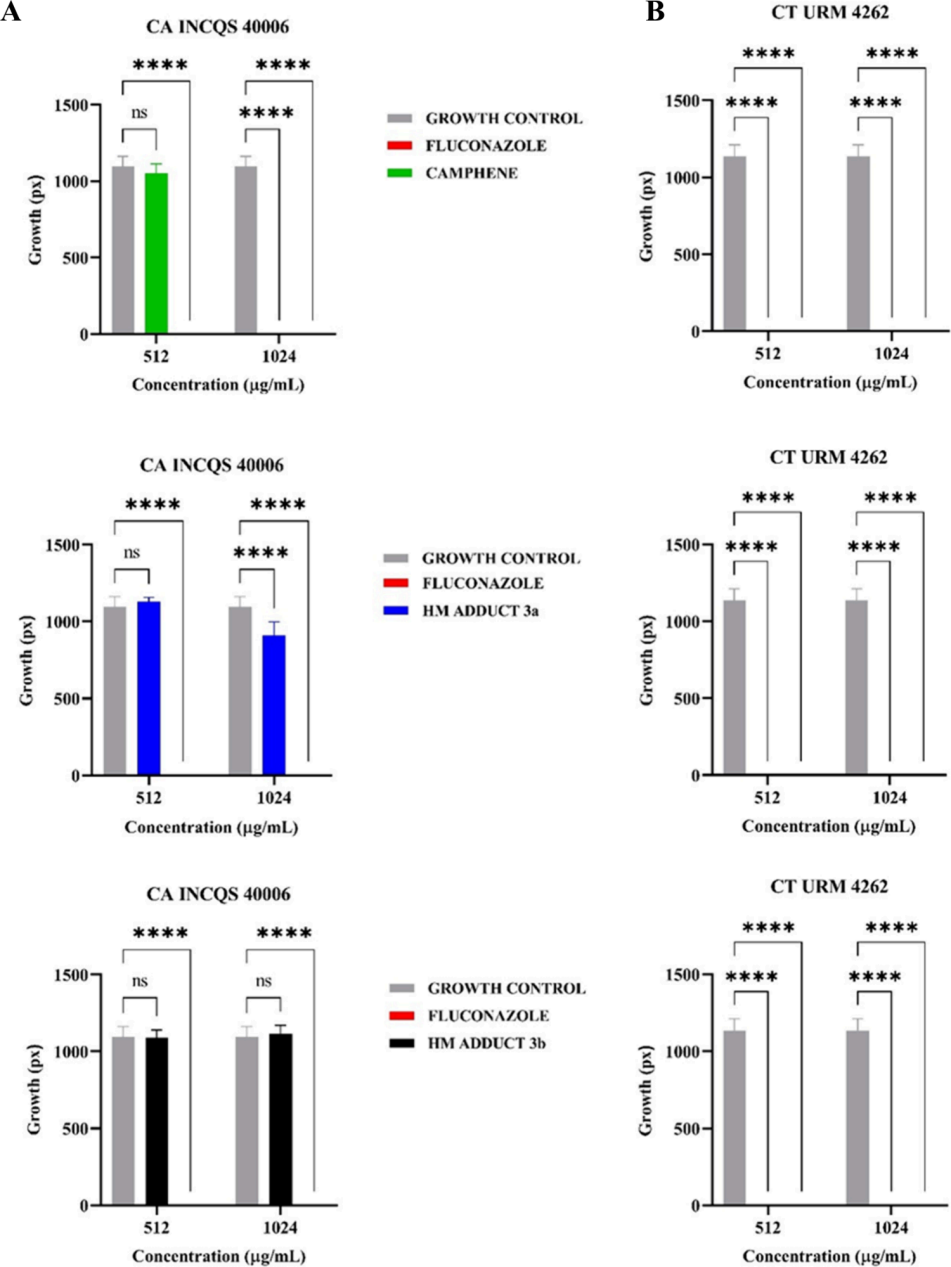
Effect of camphene, derivatives, and fluconazole on the morphological transition of *Candida albicans* and *Candida tropicalis*. (A) CA: *Candida albicans*; INCQS: National Institute for Quality Control in Health; (B) CT: *Candida tropicalis*; URM: University Recife Mycology. FCZ: Fluconazole; CAM: Camphene; HM: Heck Matsuda; and ns: not significant; ****p < 0.0001, statistical significance.

Figures 3 and 4, obtained through optical microscopy, validate the statistical results shown. The morphological transition is adequately expressed in the growth control of CA INCQS 40006 in Figure 3A. Figure 3B,C shows the results obtained by FCZ, where total inhibition of hyphal and/or pseudohyphal filaments was observed at both concentrations evaluated. CAM inhibited pleomorphism in CA INCQS 40006 in CM, as shown in Figure 3D, and slightly reduced it in CM/2 (Figure 3E). Figure 3F,G shows that HM adduct **3a** was not able to inhibit filament growth in CA INCQS 40006 at any of the concentrations, as well as HM adduct **3b**, shown in Figure 3H,I.

**Figure 3:**
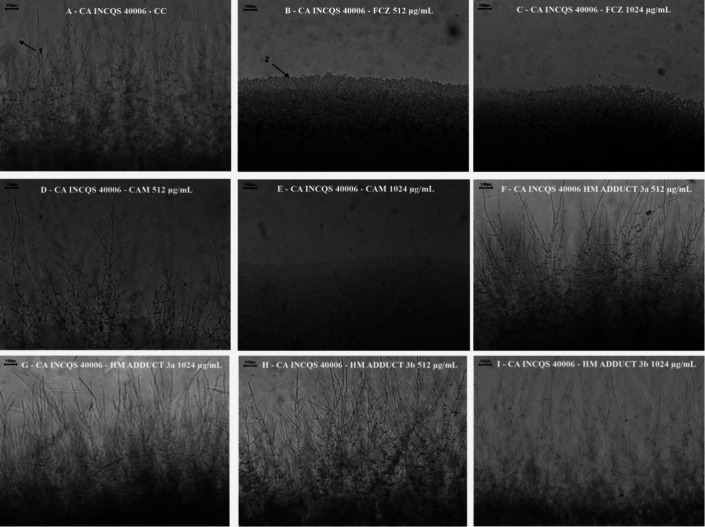
Morphological transition of *Candida albicans*. CA: *Candida albicans*; INCQS: National Institute for Quality Control in Health; CC: Growth control; FCZ: Fluconazole; CAM: Camphene; HM: Heck Matsuda. The arrows indicate filamentous structures (**1**) and yeast cells (**2**). (A) Growth control; (B,C) FCZ (512 e 1024 μg/mL); (D,E) CAM (512 e 1024 μg/mL); (F,G) HM adduct **3a** (512 e 1024 μg/mL); and (H,I) HM adduct **3b** (512 e 1024 μg/mL). The images were captured using optical microscopy at 10× magnification.

**Figure 4:**
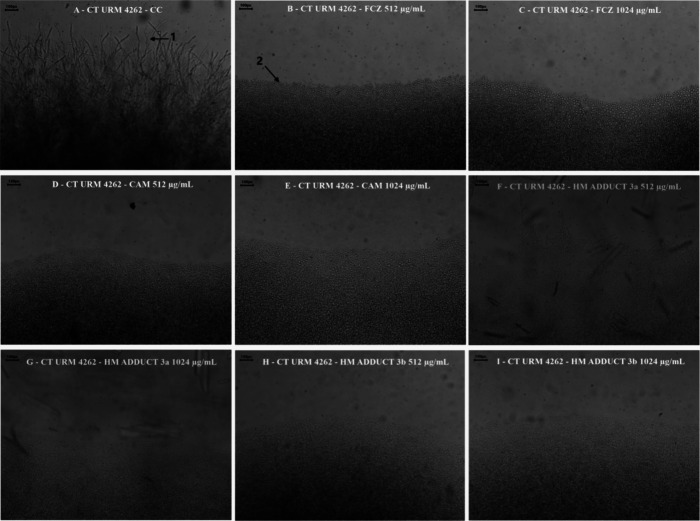
Morphological transition of *Candida tropicalis*. CT: *Candida tropicalis*; URM: University Recife Mycology; CC: Growth control; FCZ: Fluconazole; CAM: Camphene; HM: Heck Matsuda. The arrows indicate filamentous structures (**1**) and yeast cells (**2**). (A) Growth control; (B,C) FCZ (512 e 1024 μg/mL); (D,E) CAM (512 e 1024 μg/mL); (F,G) HM adduct **3a** (512 e 1024 μg/mL); and (H,I) HM adduct **3b** (512 e 1024 μg/mL). The images were captured using optical microscopy at 10× magnification.

Figure 4A shows the growth control of CT URM 4262, where the emission of filamentous structures is evident. Figure 4B,C demonstrates the inhibition of filaments by the action of FCZ. The total inhibition of the growth of these structures by the action of the compounds at both CM and CM/2 concentrations is visualized in Figure 4D–I.

## 4 Discussion

### 4.1 Chemical Studies

The molecular formula of HM adduct **3a** (aryl-camphene-type derivative) was established to be C_17_H_22_O by HRMS and elemental analysis (see S.I. for details). This finding was also supported by 15 signals in the ^13^C NMR spectrum attributable to two methyl groups, one methoxy group, two olefinic carbons, three methylene groups, one aliphatic quaternary carbon, two CH aliphatic carbons, and four signals from aromatic carbons (with two signals from magnetically equivalent carbon atoms), in line with the data reported in the literature for 8-substituted-camphene type compounds.^26^ The 1D NMR data for **3a** showed many similarities to the data for camphene (**1**), except for the disappearance of one of the signals related to the H-8 olefinic hydrogens, which was replaced at position C-8 by the *p*-methoxyphenyl group, giving rise to the appearance of signs of aromatic and methoxyl hydrogens. The presence of aromatic and olefinic groups was also confirmed by IR spectroscopy of derivative **3a** (υ = 3038, 2952, 1509, and 1462 cm^–1^).

The regiochemistry and *E*-geometry of the double bond in the aryl-camphene-type derivative **3a** were unambiguously assigned through comprehensive NMR spectroscopic investigation using 2D methods, and this assignment was extended to the other HM adducts (**3b**–**g**). A combination of H,H–COSY, HSQC, HMBC, and NOESY experiments allowed a complete signal assignment for compounds **3a** (Figure 5a). The numbering scheme refers to the signal assignment used in the Supporting Information (Table S2) and in the discussion. The HMBC long-range correlation between H-8 (δ_H_ 5.95) and C-12 (δ_C_ 157.49), as well as between H-8 (δ_H_ 5.95) and C-10 (δ_C_ 29.23), established connectivity and consequently regiochemistry (Figure 5b). Finally, the *E*-geometry was determined based on the nuclear Overhauser effect (NOE) correlations observed in the NOESY spectrum between the vinylic hydrogen, H-8 (δ_H_ 5.95), and the methyl group closest and most unshielded by the anisotropic effect of the double bond, H-10 (δ_H_ 1.12), as well as between the *ortho*-hydrogens of the aromatic ring, H-12 (δ_H_ 7.18–7.21), with both vinylic hydrogen, H-8 (δ_H_ 5.95), and the most unshielded hydrogen in the bridgehead, H-1 (δ_H_ 1.94–1.95) (Figure 5c).

**Figure 5:**
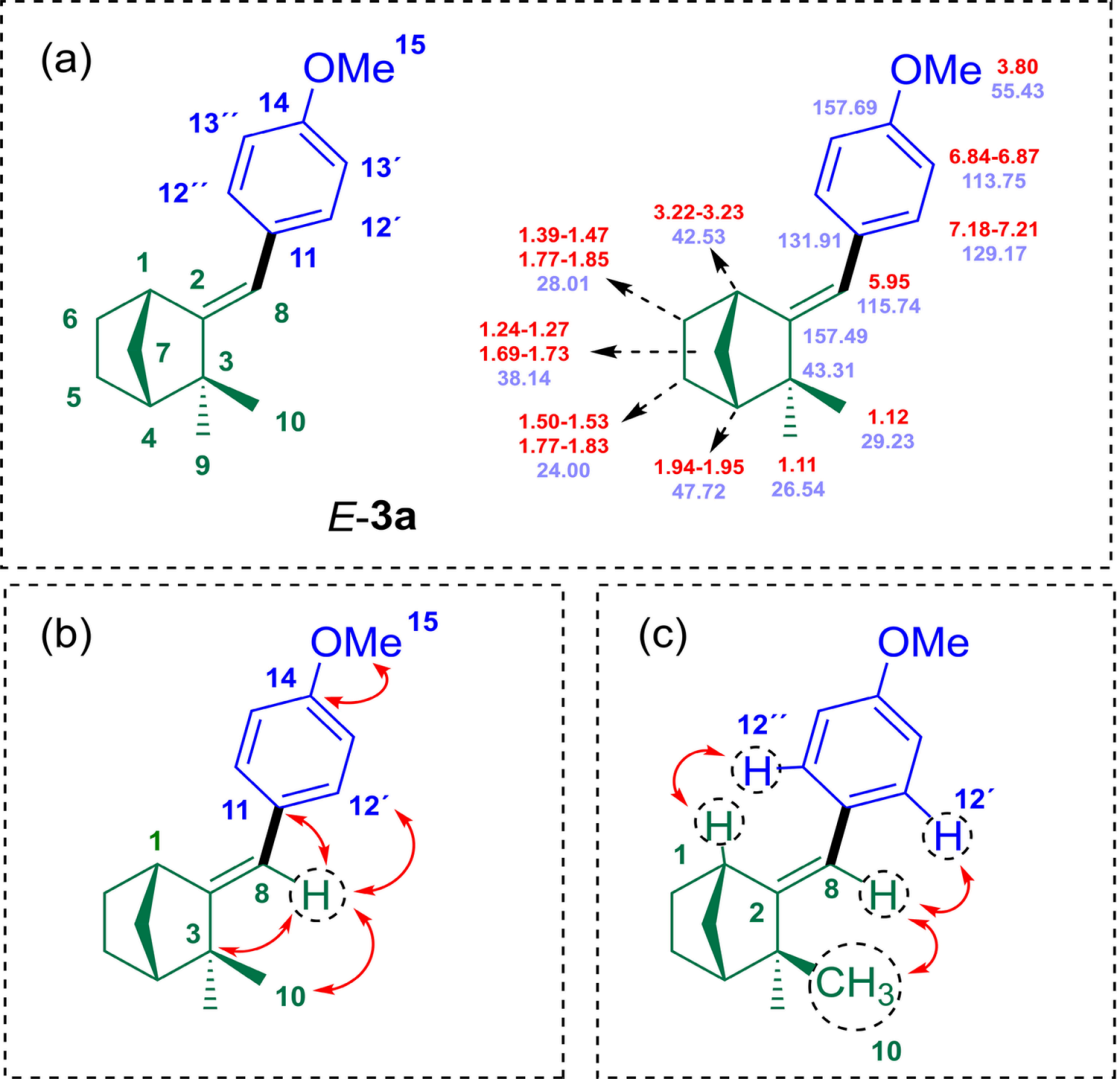
Numbering scheme and complete assignment of ^1^H (red) and ^13^C (blue) NMR signals (a). Key HMBC (b) and NOESY (c) correlations observed for HM adduct **3a**.

A plausible rationale for the HM arylation of camphene (**1**) is proposed in Scheme 1. The mechanism begins with the oxidative addition of zerovalent Pd species [Pd(0)­L_n_] to arenediazonium salt **2**, resulting in the extrusion of molecular nitrogen and the formation of cationic arene-palladium reactive intermediate **A**. Based on the regiochemistry and stereoselectivity observed in the (*E*)-**3**-type HM adduct, it can be proposed that the arene-palladium intermediate approaches the olefin from the less sterically congested face of the bicyclic system, the minor bridge, as shown in representation **B** of the proposed catalytic cycle system. This approach should minimize steric interactions and favor the appropriate arrangement for the stereoselective migratory insertion stage, as indicated in **C**. Therefore, in the β-syn-elimination stage, the aryl group should preferentially assume a position that minimizes steric interactions and allows *syn*-alignment between palladium and the benzylic hydrogen (conformer **D**), which favors the β-*syn*-elimination step and produces HM adduct **3** with an *E*-geometry.

**Scheme 1:**
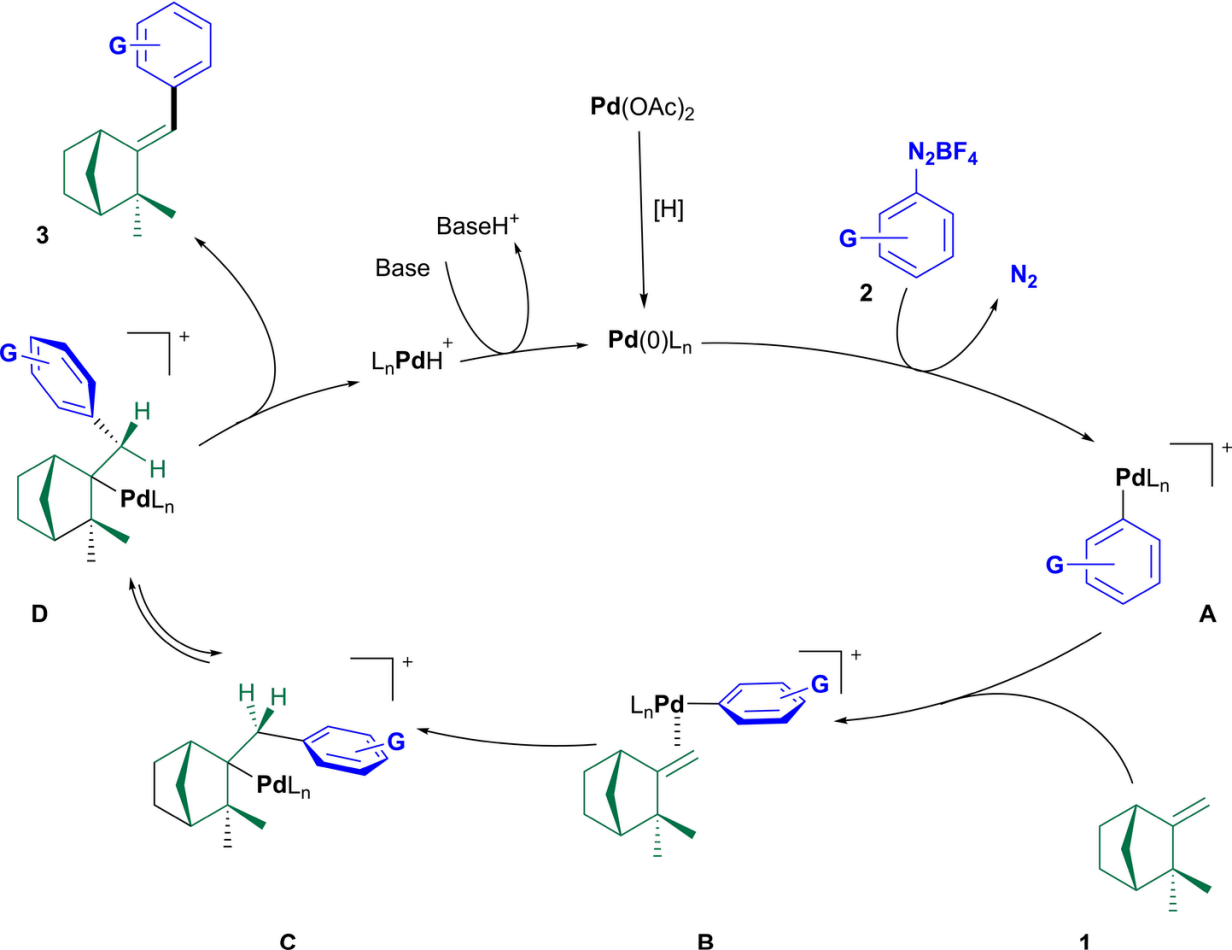
Rationale for Formation of HM Adducts (*E*)-**3a**–**g**

### 4.2 Antifungal Tests

Previous studies have demonstrated the antimicrobial properties of both camphene itself and some camphene-based chemical derivatives against infectious microorganisms.^20,33,34^ Based on these properties, we decided to evaluate the antifungal potential of camphene (**1**) and its HM adducts **3a**–**b** against strains of *Candida albicans* and *Candida tropicalis*. The antifungal evaluation was limited to compounds **3a**–**b** due to their availability and initial screening results, as these derivatives were obtained in amounts sufficient for reproducible biological assays, whereas compounds **3c**–**g** were prepared mainly to showcase the scope of the Heck–Matsuda methodology and were available only in limited quantities.

*Candida albicans* and *C. tropicalis* are considered by the WHO to be priority fungal pathogens in the context of threats to human health. They are characterized as having a critical and high pathogenicity index, respectively.^35^ When evaluating the concentration capable of inhibiting the growth of fungal cells of *C. albicans* and *C. tropicalis* by 50%, camphene **1** (CAM) showed a superior effect to fluconazole (FCZ) against *C. albicans*. At the same time, the action of its HM adducts **3a**–**b** was inferior to that of this drug. Against *C. tropicalis*, both camphene (CAM, **1**) and its derivatives (**3a**–**b**) were significantly less effective than FCZ. Despite these results, it is necessary to consider that the concentration values obtained are significantly relevant within the clinical context.^36^

The isolated action of camphene against *Candida* spp. has already been reported in some studies. Salgueiro and co-workers, for example, using the broth microdilution technique,^37^ evaluated the antifungal potential of camphene obtained from the plants *Thymus camphoratus* and *T. carnosus* against *C. albicans*, *C. guilliermondii*, *C. krusei* (*Pichia kudriavzevii*), *C. parapsilosis*, and *C. tropicalis*, obtaining MIC and MFC ranging from 8.9 to 17.8 mg/mL (8.900–17.800 μg/mL). The isolated action of camphene extracted from the essential oil of *Salvia lavandulifolia* was also evaluated against *C. albicans*,^34^ where, according to the authors, camphene showed an MIC and MFC >2.0 mg/mL (>2000 μg/mL). Comparatively, in our studies, the isolated action of camphene **1** (CAM) and HM adducts **3a**–**b** showed MFC ≥1024 μg/mL for *C. albicans* and *C. tropicalis*. These results, particularly regarding MFC, suggest an action similar to that reported previously in the literature.^34,37^

It is worth noting that in our assessment, the MFC determination was also obtained by combining the compounds with fluconazole (FCZ), indicating a strong interaction between the compounds (**1**, **3a**–**b**) and this drug. In this perspective, Jiang and co-workers point out that combination therapy proves to be an effective strategy in the search for new synergistic targets that can increase the efficacy of FCZ and decrease fungal resistance,^38^ considering that the fungistatic character and the continuous and prophylactic use of this drug are determining factors for the increase in resistance to this drug.^39^

Still considering this perspective, Yan and co-workers state that several small molecules, which have been shown to have no or weak antifungal activity,^40^ effectively restore the efficacy of this drug’s antifungal activity when combined with FCZ. In addition to restoring antifungal action, the combination can also potentiate its effect.^41^ In this way, the results found in this study seem to be in line with this statement, where the association CAM + FCZ and HM adduct **3a** + FCZ significantly reduced the IC_50_ of FCZ and the compounds against *C. albicans* and *C. tropicalis*. The combination HM adduct **3b** + FCZ showed a slight potentiation of the effect against *C. tropicalis* and antagonized the effect against *C. albicans*.

Some studies have also shown synergism in the combination of CAM (**1**) and FCZ against planktonic growth and biofilm formation in *C. albicans*.^42^ Nevertheless, it is noteworthy that no studies in the literature report the analysis of the combination of CAM (**1**) and FCZ against *C. tropicalis*. Moreover, to the best of our knowledge, this is also the first investigation to evaluate the action of HM adducts **3a** and **3b** on *Candida* strains intrinsically and in combination with FCZ.

Notably, a key virulence factor in *C. albicans* and *C. tropicalis* is the ability to switch between morphological forms, such as yeast, hyphae, or pseudohyphae.^43^ Environmental cues like temperature, CO_2_ level, pH, *N*-acetylglucosamine, and nutrition trigger this structural plasticity. A network of signaling pathways regulates these stimuli, playing an essential role in the morphological transition of *Candida* species.^44–46^

In the present study, the morphological transition of *C. albicans* and *C. tropicalis* was completely inhibited by the action of FCZ at the concentrations evaluated (1024 and 512 μg/mL). FCZ acts by inhibiting the synthesis of ergosterol – an essential component for maintaining membrane integrity and for hyphal formation.^38,48^ It has been recognized that damage to the genes involved in this process results in inhibition of the growth of these filamentous structures.^38,47,48^ Furthermore, FCZ is also capable of causing mitochondrial dysfunction in *Candida* cells, impairing the virulence of these species, since mitochondrial maintenance is essential for the yeast-hypha transition.^49,50^ Of note, CAM (**1**) inhibited the emission of hyphae and/or pseudohyphae in *C. albicans* only at the highest concentration evaluated, and its HM adducts **3a**–**b** did not significantly inhibit or reduce these filamentous structures. Against *C. tropicalis*, the compounds completely inhibited the growth of hyphae and or pseudohyphae at both concentrations tested.

The inhibitory action of camphene (CAM, **1**) and its HM adducts **3a**–**b** on the morphological transition of *C. tropicalis* may be associated with damage to the membrane integrity of this species due to the terpenic nature of these compounds, a class of metabolites well-known to cause damage to the structure and functions of the *Candida* cell membrane.^51^ The results from the morphological transition test showed that the compounds had varying effects on the strains evaluated. These findings align with recent studies that highlight that, despite the similarities between *C. albicans* and *C. tropicalis*, these species exhibit distinct behaviors and filamentous responses.^52^ This behavior has been related to factors such as intrinsic characteristics and divergent regulatory networks,^52^ which may also explain the differences observed in our results.

Karuppayil and co-workers have also reported on the inhibitory action of camphene (CAM, **1**) on the morphogenesis of *C. albicans* (3 mM–408.72 μg/mL).^42^ In addition, camphene exhibits intrinsic activity against biofilm formation with an inhibition percentage of 70% (48 mM–6.539.52 μg/mL) and, when combined with FCZ, achieves 80% inhibition (1.5 mM–204.36 μg/mL).^42^ Biofilms are microbial communities that adhere to surfaces and host tissue, representing one of the main virulence factors contributing to the pathogenesis of *Candida*; filamentation is one of the main steps in the formation of these biofilms.^53^ In the wake of these studies, Salgueiro and co-workers also evaluated the action of camphene on germ tube formation in *C. albicans*, an essential stage for filamentation.^37^ However, the isolated action of this compound did not achieve the same efficacy (91% inhibition at 0.28 mg/mL–280 μg/mL) compared to the essential oils of the plants from which it was extracted, *T. camphoratus* (about 91% at 0.07 mg/mL–70 μg/mL) and *T. carnosus* (about 98% inhibition at 0.07 mg/mL–70 μg/mL).^37^

Overall, both alone and combined with fluconazole (FCZ), camphene (CAM, **1**) showed superior antifungal activity compared to HM adducts **3a**–**b**. These observations align consistently with the analysis by Silva and co-workers,^54^ which indicates that different antifungal analogues of the same terpene class can exert distinct effects on the same cell, depending on their mechanisms of action. In addition, it is essential to note that camphene (CAM, **1**) has a comparatively smaller molecular volume and lower molecular mass than the HM adducts **3a**–**b**. Therefore, it may interact with and cross the fungal cell membrane more easily. Finally, the monoterpene nature of camphene could also explain this result, since, according to the literature, monoterpenes act on the fungal cell by destabilizing the cell membrane via interaction with ergosterol, increasing its fluidity and permeability.^55,56^

Furthermore, based on the distinguishable antifungal activities between camphene (CAM, **1**) and the HM adducts **3a**–**b** investigated in this study, it is also possible to suggest that adding an olefin-conjugated aryl group at the C-8 position of the camphene scaffold is detrimental to its antifungal efficacy against the *Candida* spp. strains evaluated. Inserting the aryl group into the camphene scaffold may have led to several effects, including increased molecular size, larger steric hindrance, altered metabolic stability in a fungal environment, and changes in solubility and permeability due to the aryl-hydrophobic interactions. These effects, alone or together, likely contributed to the reduced antifungal activity seen for the HM adducts **3a**–**b**.

Taken together, these results clearly indicated that the 8-arylidene portion in the camphene scaffold plays an important role in the decrease in antifungal activity observed for the tested aryl-labdane-type derivatives (HM adducts **3a**–**b**) compared to the natural precursor (CAM, **1**) lacking geometric isomerism. Therefore, some general considerations regarding the structure–activity relationship (SAR) between compounds **1** and **3a**–**b** may be rationalized. First, it can be noticed that geometric isomerism imposed on the unsaturation at C-8 of the camphene structure in the synthesized HM adducts **3a**–**b** apparently has a profound impact on the antifungal potential, suggesting that the disubstituted double bond in the structure of camphene may play an essential role in the lipophilicity profile and enhancement of cellular uptake for triggering biological activity. Second, the increase in molecular volume imposed on the camphene structural framework may have altered the appropriate lipophilicity profile, thereby limiting the permeability of these HM adducts through the fungal cell membrane. Despite this, in a comparative analysis of derivatives, HM adduct **3a**, with a *p*-methoxy electron-donating group on the aryl group, showed the best antifungal activity against *C. albicans* and *C. tropicalis*. This last observation suggests that further structural refinements aimed at synthesizing novel 8-aryl-camphene-type derivatives (HM adducts) with electron-rich aryl groups may constitute a window of opportunity for the development of new antifungal lead compounds, and further studies in this direction are now underway in our research group. Finally, but still worth noting, the insertion of aryl-type substituents bearing different electron-donating and electron-withdrawing groups at various positions (ortho, meta, and para) within a privileged scaffold remains a promising strategy for the development of new antifungal agents, as evidenced by studies on SAR in this field, well reported in the literature.^57,58^

Despite this reduction compared to the camphene molecule itself, the results obtained by HM adducts **3a**–**b** may still be considered relevant. The antifungal activity exhibited by these compounds may be related to the presence of specific functional groups, such as methoxy and nitro aryl substituents. According to Korol et al.,^59^ the presence of aryl substituents such as methoxy and nitro in particular positions of the aryl moiety in antifungal lead compounds can significantly increase the inhibition of *Candida* fungal growth. In line with this perspective, Sousa and co-workers reported that antifungal compounds based on benzoic acid,^60^ containing nitro groups linked to an aromatic ring, exhibited antifungal activity against *C. albicans*, *C. tropicalis*, and *C. krusei*. Likewise, Khabnadideh and co-workers reported that the addition of a methoxy group to aryl moieties of azole derivatives was decisive for antifungal efficacy.^61^

## 5 Conclusions

In summary, we have revisited and extended the Heck–Matsuda (HM) cross-coupling reactions between camphene (**1**) and arenediazonium salts (**2**) to provide aryl-camphene-type derivatives (HM adducts) with controlled regiochemistry and high stereoselectivity, using Pd­(OAc)_2_ as the catalyst, EtOH as the solvent, ZnCO_3_ as the base, and ligand-free and aerobic conditions. The developed methodology represents a contemporary approach to the late-stage structural diversification of camphene, offering products with biological potential, as explored in this work.

The results from the evaluation of antifungal activity demonstrate that camphene (CAM, **1**) had a superior antifungal effect on its HM adducts **3a**–**b** and exhibited intrinsic antifungal action against *C. albicans* at lower concentrations compared to FCZ (fluconazole). In addition to presenting fungicidal action against *C. albicans* and *C. tropicalis* when associated with FCZ. Their derivatives showed moderate intrinsic antifungal activity, but when combined with FCZ, they potentiated the action of this drug in almost all combinations. The morphological transition from yeast to hyphae was inhibited in *C. albicans* by the action of CAM at the highest concentration evaluated. Against *C. tropicalis*, this transition was completely inhibited by the action of all the compounds at the concentrations tested, indicating potential against this virulence mechanism present in *C. tropicalis*.

Therefore, the capacity to intensify the action of FCZ against *C. albicans* and *C. tropicalis*, and to inhibit the morphological transition of *C. tropicalis*, suggests the bioactive potential of these compounds, especially camphene, in developing therapeutic applications and reversing the fungal resistance of *Candida* species to fluconazole. Future studies to evaluate the cytotoxicity of the compounds, the possible mechanisms of action involved in the activities described, and, later, in vivo trials are of the utmost importance. These studies will complement this research and indicate possible pharmacological use. Furthermore, it will also be pertinent to evaluate the antifungal effect of other synthesized HM adducts that were not addressed in this antifungal investigation.

## Supplementary Material



## Notes

Experimental details for methodological optimization, materials, characterization data (analytical and spectral data), and copies of NMR (1D and 2D experiments) and HRMS spectra for the compounds **3a**–**g** (PDF)
