# Supplementary material for: Antifungal Efficacy against Candida spp. Strains and Synthesis via Heck–Matsuda Arylation of Aryl-Camphene-Type Derivatives
Source: ACS Omega. 2026 Mar 20;11(12):18939–51. doi: 10.1021/acsomega.5c11226 (PMC13044658; doi:10.1021/acsomega.5c11226)
Supplement: Supplementary file 1 [file ao5c11226_si_001.pdf]

SUPPORTING INFORMATION

Antifungal efficacy against Candida spp. strains and synthesis via Heck-Matsuda arylation of aryl-camphene-type derivatives

Naiza Saraiva Farias[1], Laryssa de Souza-Salgado[2], Francisco Bernardo de Barros[1], Maria Alicy Neres de Oliveira[1], Márcia Jordana Ferreira Macedo[1], Francildo dos Santos Souza[1], João Pedro Alves Torres[2], Henrique Douglas Melo Coutinho[1], Jailton De Souza-Ferrari[2, *], Maria Audilene de Freitas[1, *], and Maria Flaviana Bezerra Morais-Braga[1, *]

[b] *Department of Biological Chemistry, Regional University of Cariri, R. Cel. Antonio Luis, Crato, Ceará, 63105-000, Brazil*

[2] *Department of Chemistry, Federal University of Paraiba (UFPB), Campus I, João Pessoa, Paraíba, 58051-900, Brazil*

*To whom correspondence should be addressed:

jferrari@quimica.ufpb.br audbiologa@hotmail.com flaviana.morais@urca.br

SI consists of 25 pages, 02 tables, and 28 figures.

TABLE OF CONTENTS

1.	**General Information ………………………………………………………………………**	S3-S4
2.	**Optimization of the Heck-Matsuda reaction conditions …………….…………….…………**	S5-S5
3.	**1D and 2D NMR data for HM adduct 3a in $CDCl_3$**	S6-S6
4.	**Analytical and spectral data of the aryl-camphene-type derivatives …….……….**	S7-S10
5.	**Copies of HRMS, 1D and 2D NMR spectra ……………………….………………………**	S11-S24
6.	**Supplementary References ……………..………………………………………………**	S25-S25

1. General Information

All reagents and solvents were purchased from commercial sources; they were analytically pure and used without further purification. Room temperature (rt) refers to a standard 25°C. All reactions were stirred with teflon-coated magnetic stir bars and were monitored by thin-layer chromatography (TLC). All the reactions were carried out in 10-mL vessels under air (open-vessel). Analytical TLC experiments were performed on Macherey-Nagel TLC Silica gel (ALUGRAM Xtra Sil G/UV_{254}) precoated aluminum plates (0.20 mm thickness) and visualized using UV light (λ = 254 nm). Eluent or solvent compositions are given in (v/v). All evaporations were performed under reduced pressure on rotary evaporators with bath temperatures not exceeding 45 °C. Purifications of compounds were carried out by flash column chromatography (CC) using Neon silica gel (230−400 mesh) and employing the chromatography procedure described in the literature by Still and coworkers [1]. Purified compounds were further dried under high vacuum (0.010–0.005 mbar). Yields refer to chromatographically purified and spectroscopically (^{1}H and ^{13}C NMR) pure compounds. Temperatures above room temperature were maintained using a mineral oil bath on a hot plate.

All aryldiazonium salts employed in this research are well-known and synthesized according to established protocols in the literature by diazotization of aniline and other appropriate aromatic amines [2-3].

Melting points were recorded by using a GEHAK 1.01 PF1500 apparatus and are uncorrected. Combustion elemental analyses were performed using a Perkin-Elmer model CHN 2400 instrument.

Fourier-transform infrared (FTIR) spectroscopy was performed on an IR Prestige-21 FTIR (Shimadzu) spectrophotometer using attenuated total reflectance (ATR), and the spectra are reported in cm^{-1}. High-resolution mass spectrometry (HRMS) analyses were performed on a microTOFII ESI-TOF mass spectrometer (Bruker) or in the LCMS-9050 APCI-TOF (Shimadzu). The 1D and 2D NMR (nuclear magnetic resonance) spectra used to determine the identity and purity of synthesized compounds were performed on Bruker AVANCE III Hb spectrometers (400 or 500 MHz for ^{1}H and 101 or 126 MHz for ^{13}C). Chemical shifts are reported in parts per million (ppm) from TMS (0 ppm) and are referenced to residual protium in the NMR solvent ($CHCl_3$ = δ 7.28 for ^{1}H NMR and 77.16 ppm for ^{13}C NMR) as an internal standard. Coupling constants (*J*) are quoted to the nearest 0.1 Hertz (Hz). The descriptions of the coupling patterns of ^{1}H NMR signals are based on the optical appearance of the signals and do not necessarily reflect the physically correct interpretation. In general, the chemical shift information refers to the center of the signal. In the case of multiplets, intervals are given. Abbreviations to denote the multiplicity of a particular signal are s (singlet), d (doublet), t

(triplet), q (quartet), dd (double doublet), m (multiplet), and sl (signal large).

The synthesized compounds **3a-g** were at least 95% pure, as determined by CHN combustion elemental analyses (results within 0.4% of theoretical values).

2. Optimization of the Heck-Matsuda reaction conditions

Table S1 – Optimization studies of the Heck-Matsuda reaction of camphene (**1**)

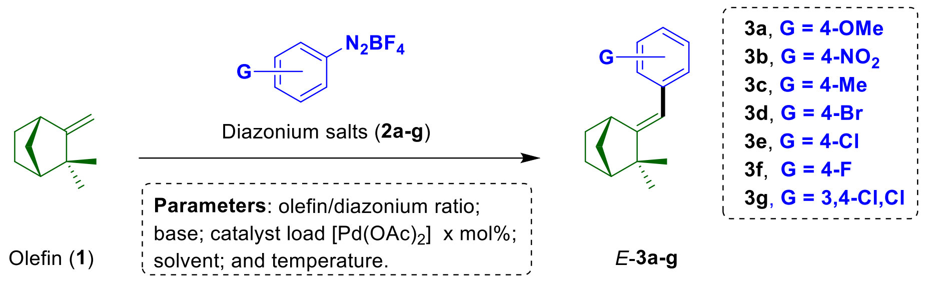

Entry	Olefin:Diazoninum (equiv.)	Base (equiv.)	$Pd(OAc)_2$ (mol%)	Solvent	T (ºC)	t (h)	Yield (%)
1	1.5 : 1.0	-	0.9	EtOH(95%)	40	3	- (**3a**)
2	1.5 : 1.0	-	0.9	EtOH	40	3	- (**3a**)
3	1.2 :1 .0	NaOAc (1.0)	5	MeOH	rt	3	- (**3a**)
4	1.2 : 1.0	$ZnCO_3$ (1.0)	5	MeOH	rt	3	5 (**3a**)
5	1.2 : 1.0	$Zn(AcO)_2$ (1.0)	5	MeOH	rt	3	- (**3a**)
6	1.0 1.5	NaOAc (1.5)	10	MeOH	rt	1	35 (**3a**)
7	1.0 : 1.5	NaOAc (1.5)	10	EtOH	rt	1	62 (**3a**)
8	1.0 : 1.5	$ZnCO_3$ (1.5)	10	EtOH	rt	1	65 (**3a**)
9	1.0 : 1.5	$Zn(AcO)_2$ (1.5)	10	EtOH	rt	1	4 (**3a**)
10	1.0 : 1.5	NaOAc (1.5)	10	EtOH	40	1	23 (**3a**)
11	1.0 : 1.5	$CaCO_3$ (1.5)	10	EtOH	40	1	37 (**3a**)
12	1.0 : 1.5	K_2CO_3 (1.5)	10	EtOH	40	1	48 (**3a**)
13	1.0 : 1.5	$ZnCO_3$ (1.5)	10	EtOH	40	1	95 (**3a**)
14	1.0 : 1.5	$ZnCO_3$ (1.5)	5	EtOH	40	1	53 (**3a**)
15	1.0 : 1.2	$ZnCO_3$ (1.5)	10	EtOH	40	1	73 (**3a**)
16	1.0 : 1.5	ZnCO3 (1.5)	10	EtOH	40	1	98 (**3b**)
17	1.0 : 1.5	$ZnCO_3$ (1.5)	5	EtOH	40	1	85 (**3b**)
18	1.0 : 1.5	$ZnCO_3$ (1.5)	2.5	EtOH	40	1	92 (**3b**)
19	1.0 : 1.5	$ZnCO_3$ (1.5)	10	EtOH	40	1	48 (**3c**)
20	1.0 : 1.5	$ZnCO_3$ (1.5)	5	EtOH	40	1	62 (**3c**)
21	1.0 : 1.5	$ZnCO_3$ (1.5)	10	EtOH	40	1	89 (**3d**)
22	1.0 : 1.5	$ZnCO_3$ (1.5)	5	EtOH	40	1	90 (**3d**)
23	1.0 : 1.5	$ZnCO_3$ (1.5)	10	EtOH	40	1	85 (**3e**)
24	1.0 : 1.5	$ZnCO_3$ (1.5)	5	EtOH	40	1	80 (**3e**)
25	1.0 : 1.5	$ZnCO_3$ (1.5)	10	EtOH	40	1	83 (**3f**)
26	1.0 : 1.5	$ZnCO_3$ (1.5)	5	EtOH	40	1	66 (**3f**)
27	1.0 : 1.5	$ZnCO_3$ (1.5)	10	EtOH	40	1	80 (**3g**)
28	1.0 : 1.5	$ZnCO_3$ (1.5)	5	EtOH	40	1	62 (**3g**)

3. 1D and 2D NMR data for HM adduct 3a in $CDCl_3$

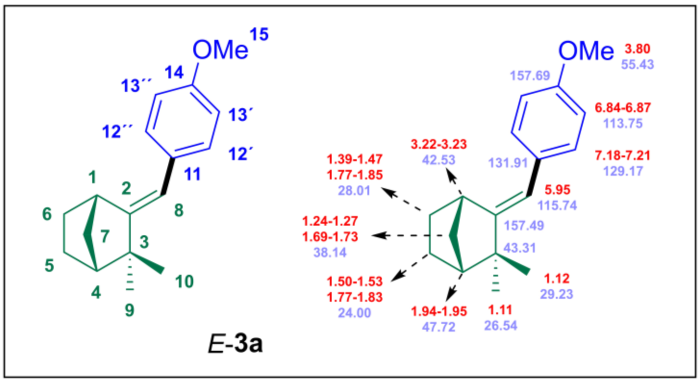

Table S2. ^{1}H (400 MHz) and ^{13}C (101 MHz) NMR Data for HM adduct **3a** in $CDCl_3$

No.	δ_C, mult*	δ_H, mult*	H,H-COSY	HMBC (H→C)	NOESY
1	42.53, CH	3.22-3.23, m	H-6, 7, 8 (^{4}J)	C-3, 4	H-4, 6, 12
2	157.49, C				
3	43.31, C				
4	47.72, CH	1.94-1.95, m	H-5, 7	C-6, 2	H-5, 9
5a	24.00, CH_2	1.50-1.53, m	H-4, 6		
5b		1.77-1.83, m	H-4, 6		
6a	28.01, CH_2	1.39-1.47, m	H-1, 5		
6b		1.77-1.85, m	H-1, 5		
7a	38.14, CH_2	1.24-1.27, m	H-1, 4		
7b		1.69-1.73, m	H-1, 4		
8	115.74, CH	5.95, s	H-1 (^{4}J)	C-3, 10, 11, 12	H-10, 12
9	26.54, CH_3	1.11, s		C-2, 3, 4	
10	29.23, CH_3	1.12, s		C-2, 3, 4	H-8
11	131.91, C				
12´	129.17, CH	7.18-7.21, m	H-13´, 13´´	C-13´, 13´´, 14	H-8, 13´, 13´´
12´´	129.17, CH	7.18-7.21, m	H-13´, 13´´	C-13´, 13´´, 14	H-8, 13´, 13´´
13´	113.75, CH	6.84-6.87, m	H-12´, 12´´	C-8, 12´, 12´´, 14	H-12´, 12´´, 15
13´´	113.75, CH	6.84-6.87, m	H-12´, 12´´	C-8, 12´, 12´´, 14	H-12´, 12´´, 15
14	157.9, C				
15	55.43, OCH_3	3.80, s		C-14	

* HSQC was used to confirm the hydrogen assignments for the hydrogen atoms attached to the carbon atoms.

4. Analytical and spectral data of the aryl-camphene-type derivatives

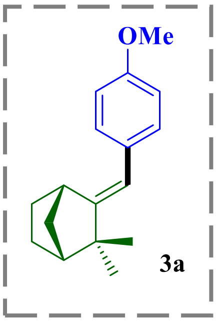

HM adduct 3a, (±)-*3-((E)-4-methoxybenzylidene)-2,2-dimethylbicyclo[2.2.1]heptane*. Prepared according to the general procedure for the HM arylation of camphene and purified by flash column chromatography (hexane/ diethyl ether 98:2, v/v) to afford **3a** as an amorphous white solid. Yield 95% (114.9 mg); mp 88–91 ºC; TLC: Rf = 0.60 (hexane/diethyl ether – 98:2); IR (ATR) ν / cm^{-1} 3010, 2952, 1888, 1509, 1462, 1358, 1243, 823; ^{1}H NMR (400 MHz, $CDCl_3$) δ 7.18-7.21 (2H, *m*); 6.84-6.87 (2H, *m*); 5.95 (1 H, *s*); 3.80 (3H, *s*); 3.22-3.23 (1H, *m*); 1.94-1.95 (1H, *m*); 1.69-1.85 (3H, *m*); 1.39-1.53 (2H, *m*); 1.24-1.27 (1H, *m*); 1.12 (3H, *s*); 1.11 (3H, *s*); ^{13}C NMR (101 MHz, $CDCl_3$) δ 157.69; 157.49; 131.91; 129.17; 115.74; 113.75; 55.43; 47.72; 43.31; 42.53; 38.14; 29.23; 28.01; 26.54; 24.00; HRMS (ESI-TOF) *m/z*, calcd. for $C_{17}H_{22}O$ $[M + H]^{+}$: 242.1671, found: 243.1743. Elemental Analysis Calcd. for $C_{17}H_{22}O$: C, 84.25; H, 9.15%, found: C, 84.27; H, 9.13%. The characterization data agree with those reported in the literature [4].

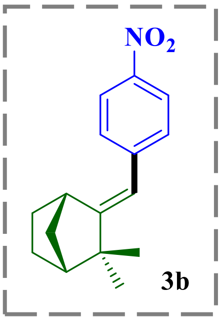

HM adduct 3b, (±)-*2,2-dimethyl-3-((E)-4-methylbenzylidene)bicyclo[2.2.1]heptane*. Prepared according to the general procedure for the HM arylation of camphene and purified by flash column chromatography (hexane/diethyl ether 98:2, v/v) to afford **3b** as an amorphous yellowish solid. Yield 96% (126.2 mg); mp 90–92 ºC; TLC: Rf = 0.43 (hexane/diethyl ether – 98:2); IR (ATR) ν / cm^{-1} 3382, 1650, 1589, 1512, 1340, 1081, 875; ^{1}H NMR (400 MHz, $CDCl_3$) δ 8.15-8.17 (2H, *m*); 7.34-7.37 (2H, *m*); 6.06 (1H, *s*); 3.23-3.24 (1H, *m*); 2.00-2.01(1H, *m*); 1.83-1.91 (1H, *m*); 1.72-1.81 (2H, *m*); 1.49-1.57 (3H, *m*); 1.39-1.46 (1H, *m*); 1.30-1.34 (1H, *m*); 1.14-1.15 (6H, *s*); ^{13}C NMR (101 MHz, $CDCl_3$) δ 164.95; 146.08; 145.52; 128.50; 123.78; 115.23; 47.49; 44.15; 42.97; 38.23; 29.04; 27.80;

26.24; 23.82; HRMS (ESI-TOF) *m/z*, calc. for $C_{16}H_{19}NO_2$ [M + H]$^+$: 257.1416, found: 258.1488. Elemental Analysis Calcd. for $C_{16}H_{19}NO_2$: C, 74.86; H, 7.44; N, 5.44%, found: C, 74.89; H, 7.42; N, 5.41%. The characterization data agree with those reported in the literature [4].

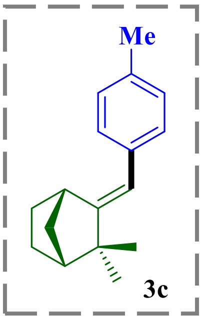

HM adduct 3c, (±)-*2,2-dimetil-3-((E)-4-metilbenzilideno)bicíclico[2.2.1]heptano*. Prepared according to the general procedure for the HM arylation of camphene and purified by flash column chromatography (hexane) to afford **3c** as a colorless viscous liquid at room temperature and a solid when refrigerated (below 20 ºC)**.** Yield 62% (54.0 mg); TLC: Rf = 0.70 (hexane/diethyl ether 98:2, v/v); IR (ATR) ν / cm^{-1} 3021, 2957, 2862, 1660, 1512, 1459, 1359, 803; ^{1}H NMR (400 MHz, $CDCl_3$) δ 7.15-7.23 (4H, *m*). 6.04 (1H, *s*). 3.29-3.31 (1H, *m*). 2.38 (3H, *s*). 2.00-2.01 (1H*, m*). 1.74-1.90 (3H, *m*). 1.45-1.58 (2H, *m*). 1.29-1.32 (1H, *m*). 1.18 (6H, *s*); ^{13}C NMR (101 MHz. $CDCl_3$) δ 158.40. 136.28. 135.18. 128.97. 128.02. 116.26. 47.73. 43.33. 42.63. 38.12. 29.25. 28.03. 26.48. 24.00. 21.22; HRMS (APCI-TOF) *m/z*, calcd. for $C_{17}H_{22}$ [M + H]$^+$: 226.3630, found: 227.1783. Elemental Analysis Calcd. for $C_{17}H_{22}$: C, 90.20; H, 9.80%, found: C, 90.23; H, 9.78%. The characterization data agree with those reported in the literature [4].

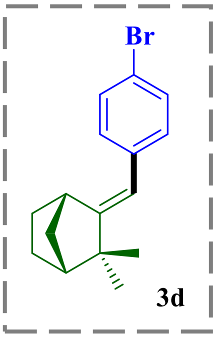

HM adduct 3d, (±)-*3-((E)-4-bromobenzylidene)-2,2-dimethylbicyclo[2.2.1]heptane*. Prepared according to the general procedure for the HM arylation of camphene and purified by flash column chromatography (hexane) and subsequently by fractional recrystallization from hexane/ethanol (4:1, v/v), resulting in **3d** as an amorphous white solid; Yield 90% (131.05 mg); mp 89–90 ºC; TLC: Rf =

0.62 (hexane); IR (ATR) ν / cm^{-1} 3028, 2964, 2857, 1684, 1653, 1620, 1483, 1465, 1357, 1065, 860, 807; ^{1}H NMR (400 MHz, $CDCl_3$) δ 7.39-7.42 (2H, *m*); 7.09-7.13 (2H, *m*); 5.93 (1H, *s*); 3.17-3.19 (1H, *m*); 1.96-1.97 (1H, *m*); 2.69-1.95 (3H, *m*); 1.36-1.56 (3H, *m*); 1.25-1.29 (1H, *m*); 1.11-1.12 (6H, *s*); ^{13}C NMR (101 MHz, $CDCl_3$) δ 161.33; 138.98; 132.21; 130.65; 120.17; 116.29; 48.53; 44.46; 43.51; 39.04; 30.05; 28.81; 27.28; 24.83; HRMS (APCI-TOF) *m/z*, calcd. $C_{16}H_{19}Br$ $[M + H]^+$: 290.0670, found: 291.0729. Elemental Analysis Calcd. for $C_{16}H_{19}Br$: C, 65.99; H, 6.58%, found: C, 65.97; H, 9.80%. The characterization data agree with those reported in the literature [4].

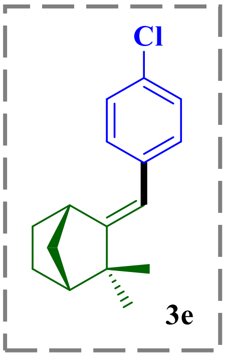

3e

HM adduct 3e, (±)-*3-((E)-4-chlorobenzylidene)-2,2-dimethylbicyclo[2.2.1]heptane*. Prepared according to the general procedure for the HM arylation of camphene and purified by flash column chromatography (hexane) and subsequently by fractional recrystallization from hexane/methanol (4:1, v/v), resulting in **3e** as an amorphous white solid; Yield 85% (105.30 mg); mp 94–95 ºC; TLC: Rf = 0.50 (hexane); IR (ATR) ν / cm^{-1} 3039, 2951, 2863, 1683, 1660, 1487, 1464, 1382, 1085, 806; ^{1}H NMR (500 MHz, $CDCl_3$) δ 7.32-7.36 (2H, *m*); 7.24-7.28 (2H, *m*); 6.04 (1H, *s*); 3.27-3.28 (1H, *d*); 2.04-2.06 (1H, *m*); 1.78-1.93 (3H, *m*); 1.55-1.64 (2H, *m*); 1.46-1.53 (1H, *m*); 1.34-1.37 (1H, *m*); 1.19-1.21 (5H, *s*); ^{13}C NMR (126 MHz, $CDCl_3$) δ 160.25, 137.62, 131.19, 129.36, 128.36, 115.35, 47.63, 43.52, 42.59, 38.13, 29.16, 27.92, 26.40, 23.92; HRMS (APCI-TOF) *m/z*, calcd. for $C_{16}H_{19}Cl$ $[M + H]^+$: 246.1175, found: 247.1235. Elemental Analysis Calcd. for $C_{16}H_{19}Cl$: C, 77.87; H, 7.76%, found: C, 77.89; H, 7.75%. The characterization data agree with those reported in the literature [4].

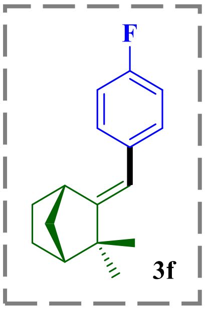

HM adduct 3f, (±)-*3-((E)-4-fluorobenzylidene)-2,2-dimethylbicyclo[2.2.1]heptane*. Prepared according to the general procedure for the HM arylation of camphene and purified by flash column chromatography (hexane) to afford **3f** as colorless oil; Yield 83% (132.30 mg); TLC: Rf = 0.55 (hexane); IR (ATR) ν / cm^{-1} 3039, 2956, 2866, 1883, 1603, 1508, 1459, 1361, 1228, 822; ^{1}H NMR (400 MHz, $CDCl_3$) δ 7.18-7.24 (2H, *m*), 6.97-7.03 (2H, *m*), 5.98 (1H, *s*), 3.19-3.21 (1H, *m*), 1.97-1.99 (1H, *m*), 1.71-1.86 (3H, *m*), 1.39-1.57 (3H, *m*), 1.27-1.30 (3H, *m*), 1.27-1.30 (1H, *m*), 1.13-1.14 (6H, *s*); ^{13}C NMR (101 MHz, $CDCl_3$) δ 162.30; 159.50 (d, *J* = 74 Hz); 135.19 (d, *J* = 3 Hz); 129.51 (d, *J* = 7 Hz); 115.24 (d, *J* = 18 Hz); 114.94; 47.56; 43.26; 42.3; 38.01; 29.08; 27.85; 26.33; 23.84; Elemental Analysis Calcd. for $C_{16}H_{19}F$: C, 83.44; H, 8.32%, found: C, 83.46; H, 8.31%. The characterization data agree with those reported in the literature.

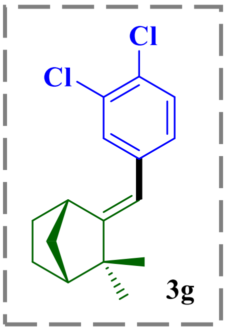

HM adduct 3g, (±)-*3-((E)-3,4-chlorobenzylidene)-2,2-dimethylbicyclo[2.2.1]heptane*. Prepared according to the general procedure for the HM arylation of camphene and purified by flash column chromatography (hexane) to afford **3g** as colorless liquid; Yield 80% (112.48mg); TLC: Rf = 0.60 (hexane); IR (ATR) ν / cm^{-1} 3052, 2956, 1892, 1580, 1546, 1461, 1378, 1026, 903, 885; ^{1}H NMR (500 MHz, $CDCl_3$) δ 7.30-7.37 (2H, *m*), 7.04-7.09 (1H, *m*), 5.90 (1H, *s*), 3.15-3.20 (1H, *m*), 1.95-1.99 (1H, *m*), 1,67-1.88 (4H, *m*), 1.36-1.60 (3H, *m*), 1.22-1.33 (1H, *m*), 1.12 (6H, s); ^{13}C NMR (126 MHz, $CDCl_3$) δ 161.66, 139.17, 132.04, 129.96, 129.70, 129.07, 127.30, 114.31, 47.43, 43.53, 42.46, 38.00, 28.99, 27.71, 26.19, 23.74; HRMS (APCI-TOF) *m/z*, calc. for $C_{16}H_{18}Cl_2$ $[M + H]^+$: 281.0786, found: 281.0845. The physicochemical and analytical data of **3g** are being described here for the first time.

5. Copies of HRMS, 1D and 2D NMR spectra

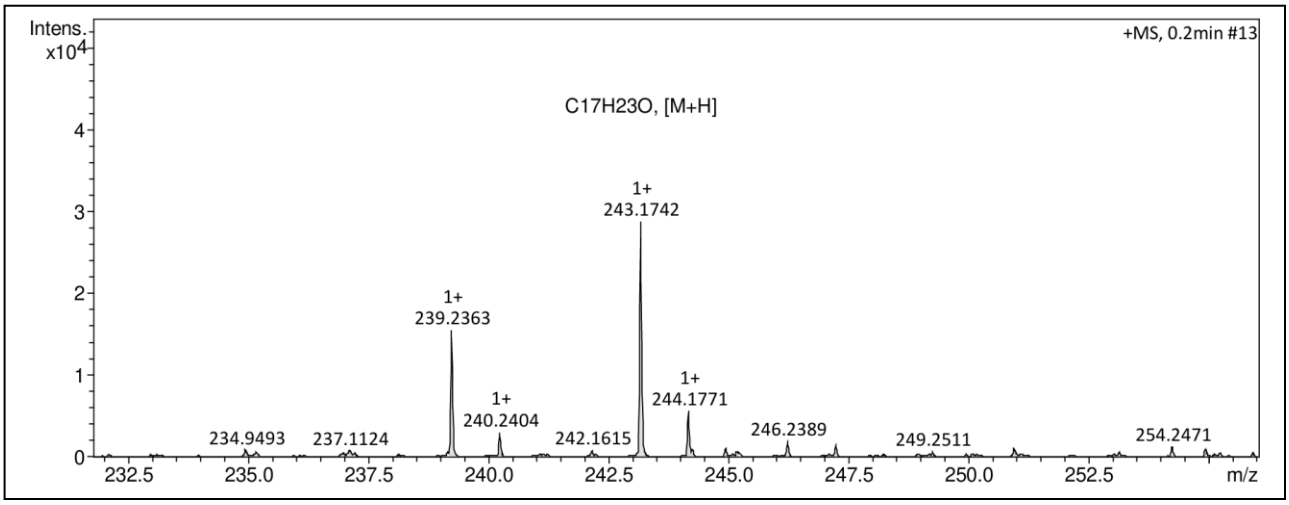

Figure S1. Mass spectrum of compound **3a**.

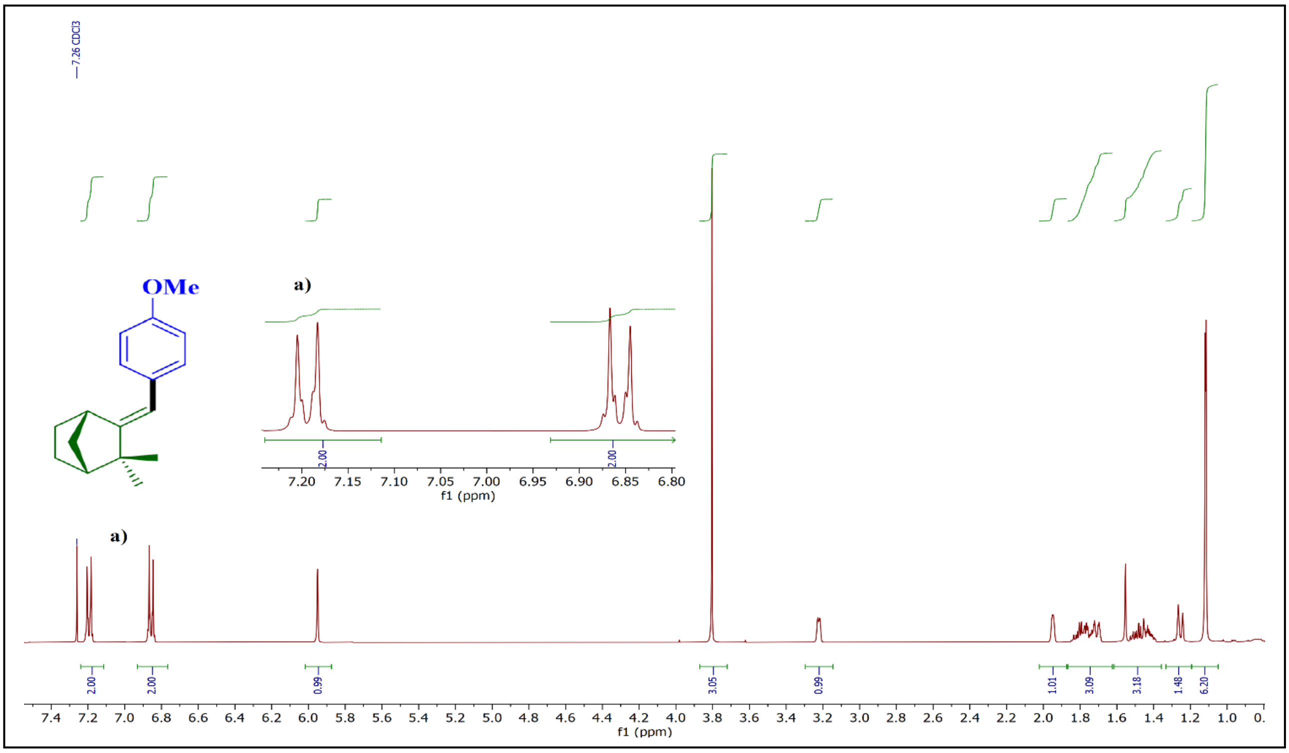

Figure S2. ^{1}H NMR spectrum (400 MHz, $CDCl_3$) of compound **3a**.

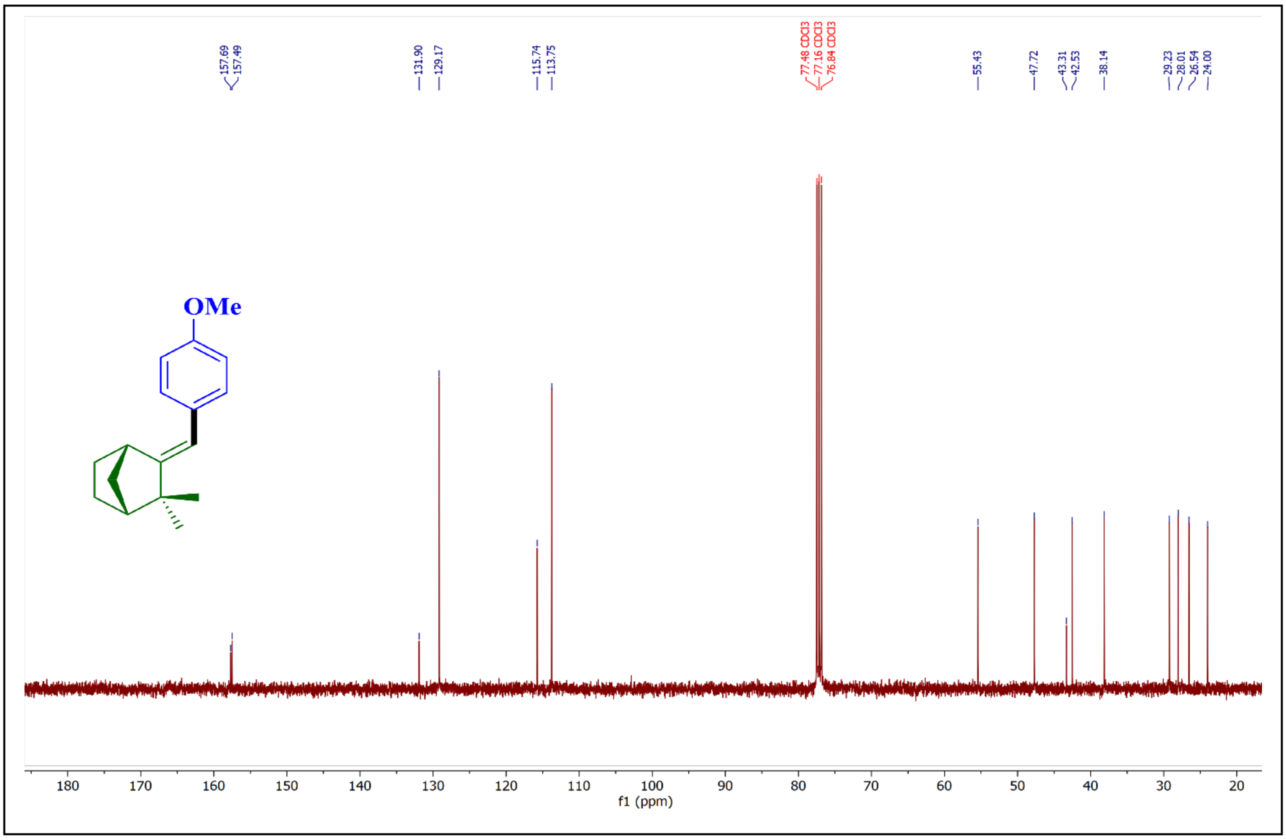

Figure S3. ^{13}C NMR spectrum (101 MHz, $CDCl_3$) of compound **3a**.

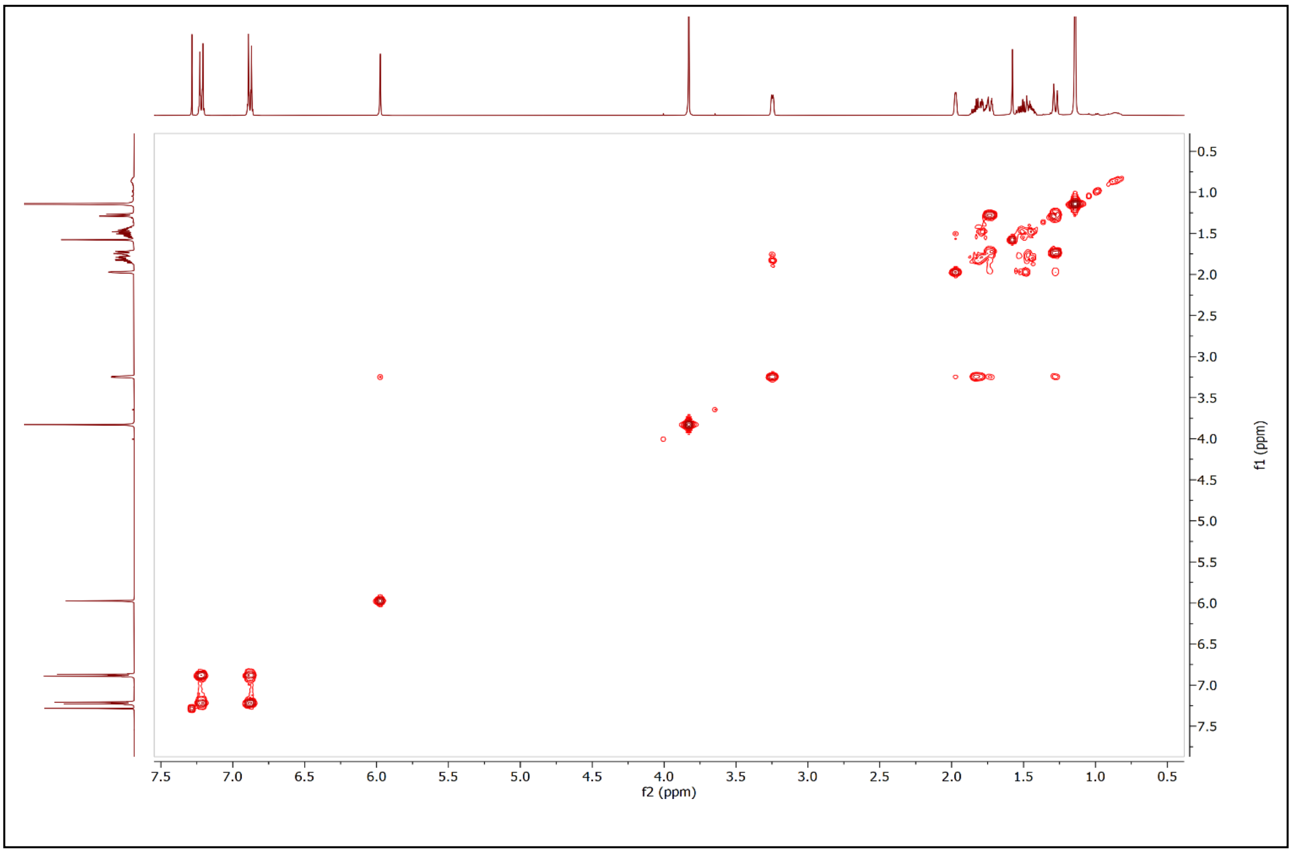

Figure S4. H,H-COSY NMR spectrum of compound **3a** in $CDCl_3$.

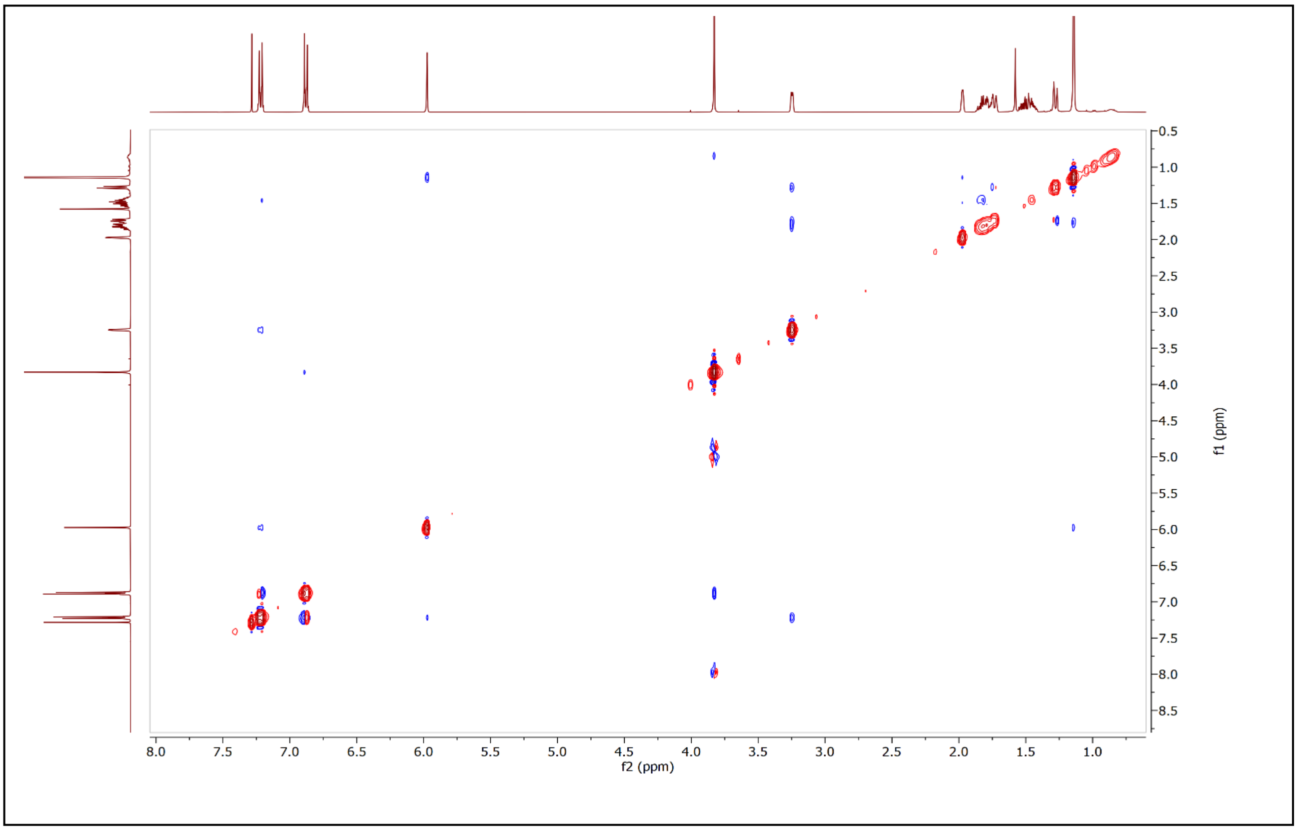

Figure S5. NOESY NMR spectrum of compound **3a** in $CDCl_3$.

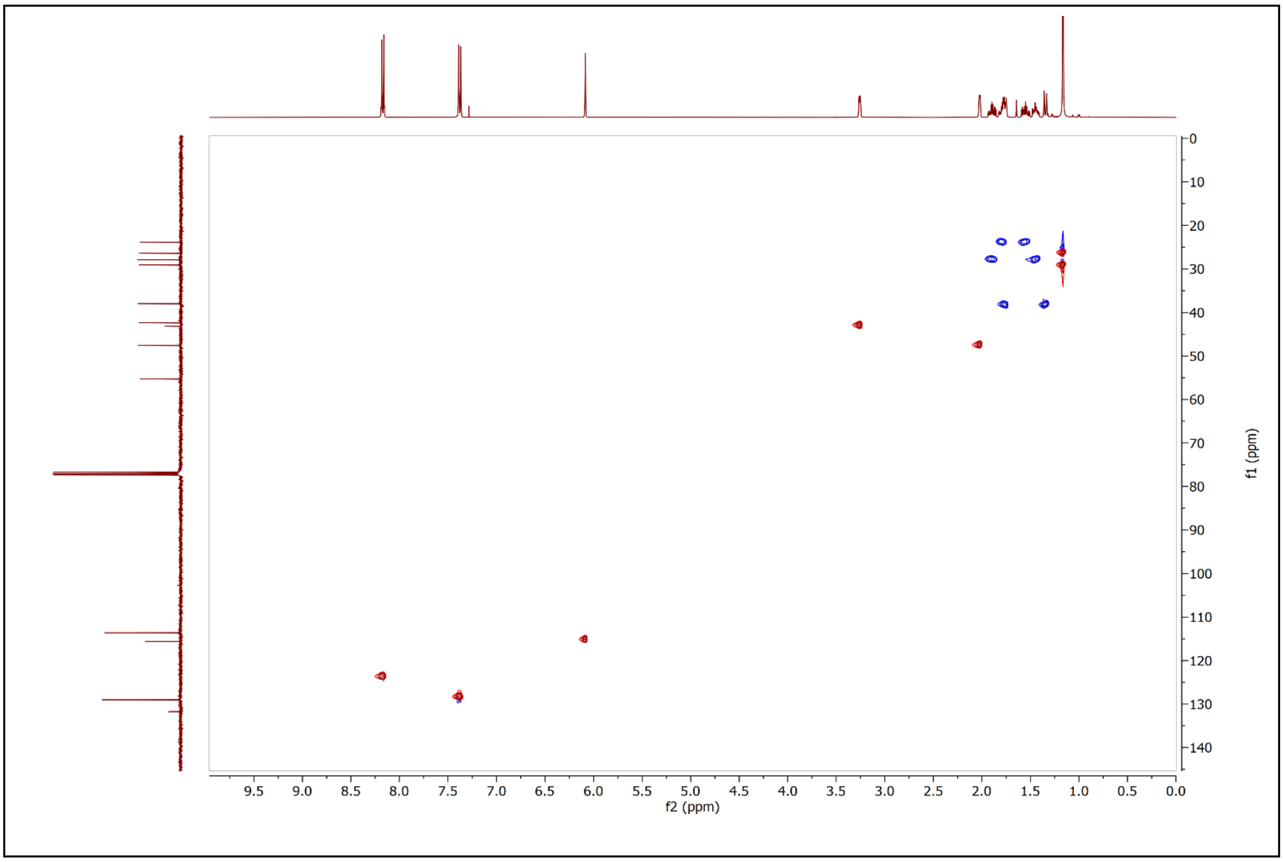

Figure S6. HMQC NMR spectrum of compound **3a** in $CDCl_3$.

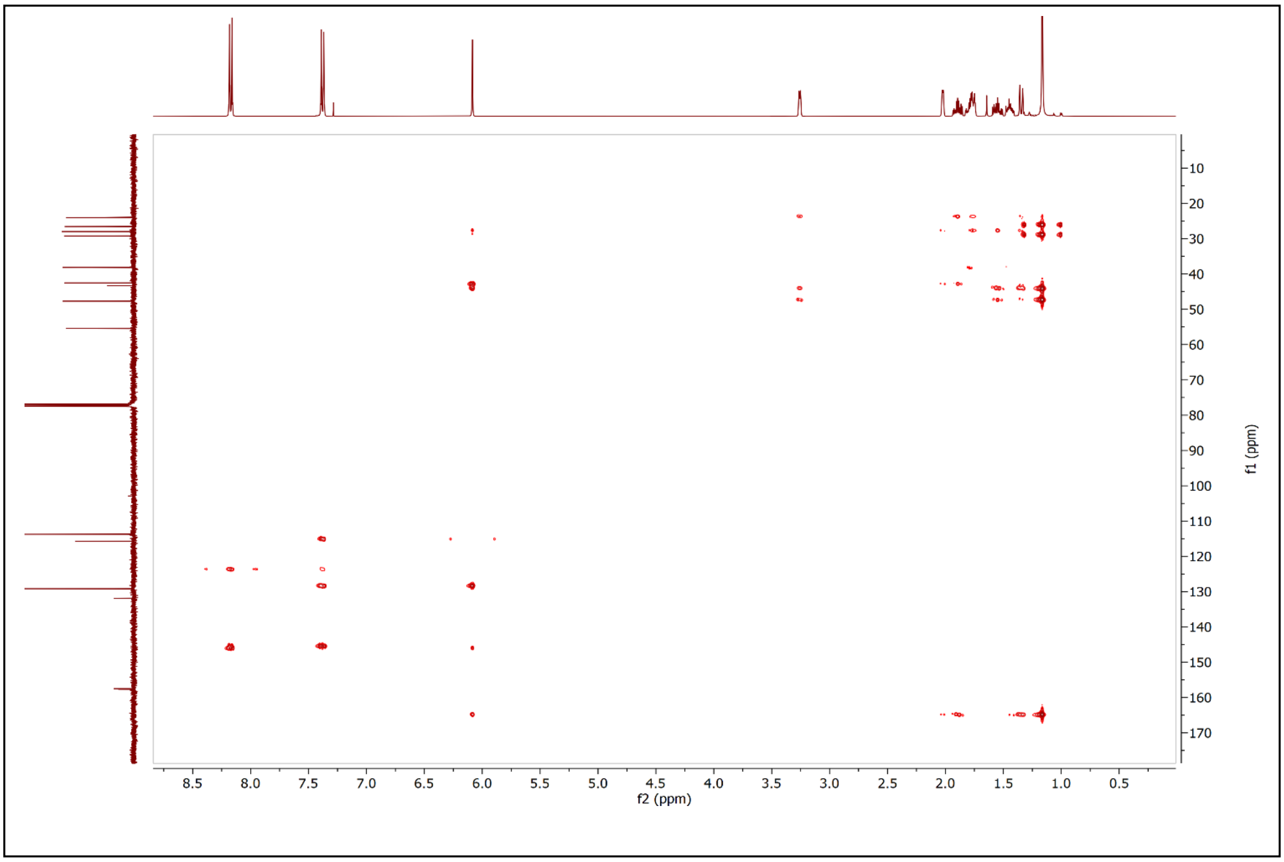

Figure S7. HMBC NMR spectrum of compound **3a** in $CDCl_3$.

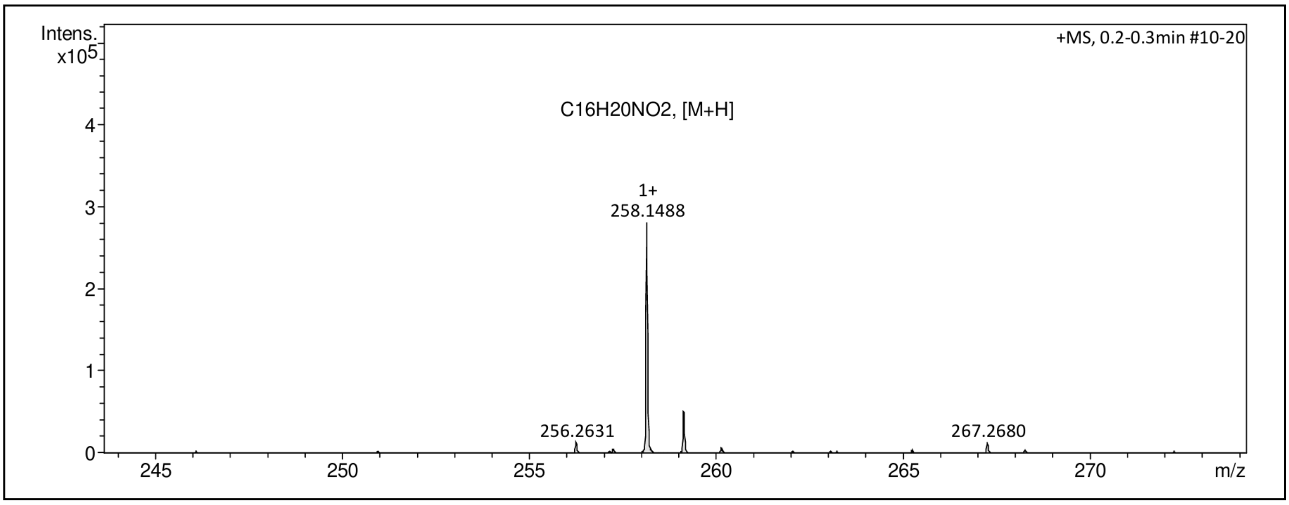

Figure S8. Mass spectrum of compound **3b**.

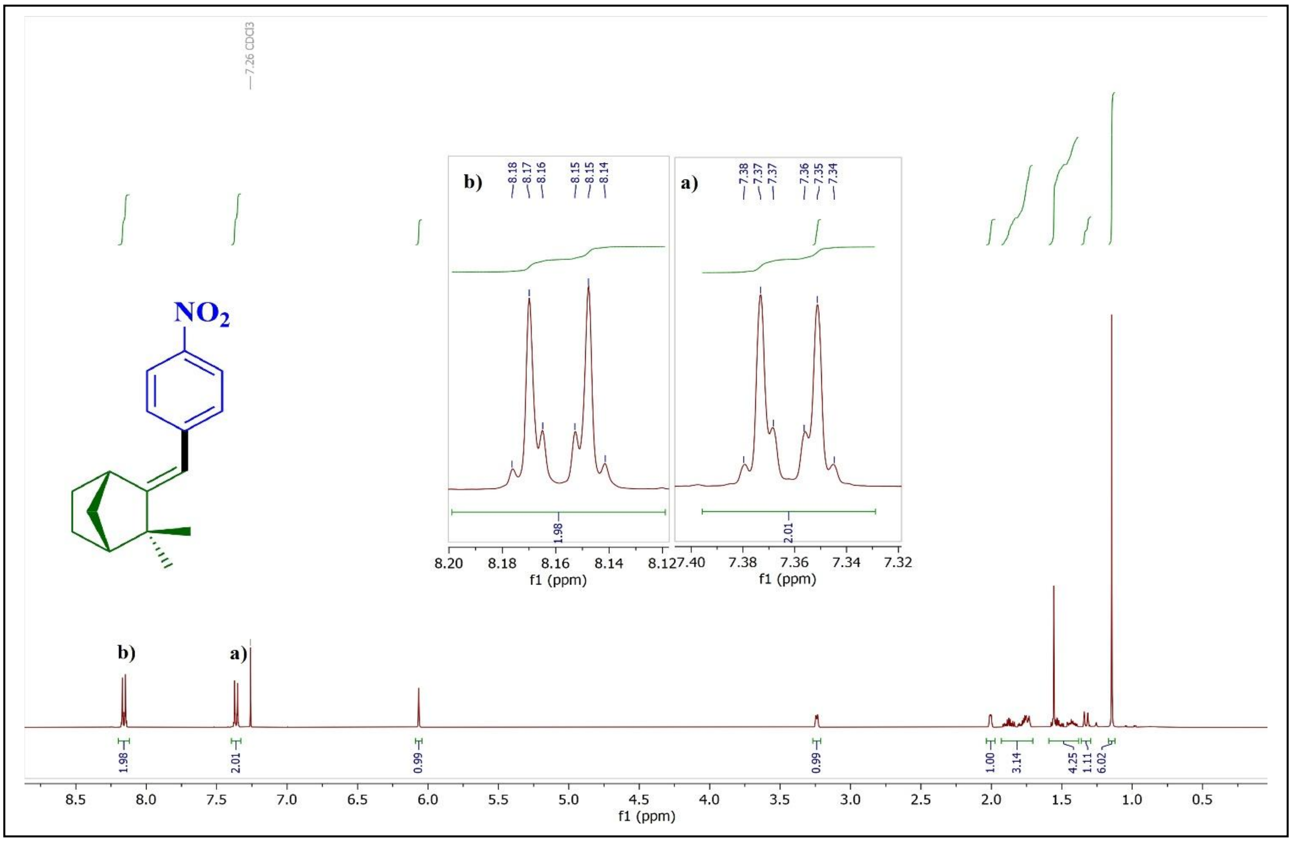

Figure S9. ^{1}H NMR spectrum (400 MHz, $CDCl_3$) of compound **3b.**

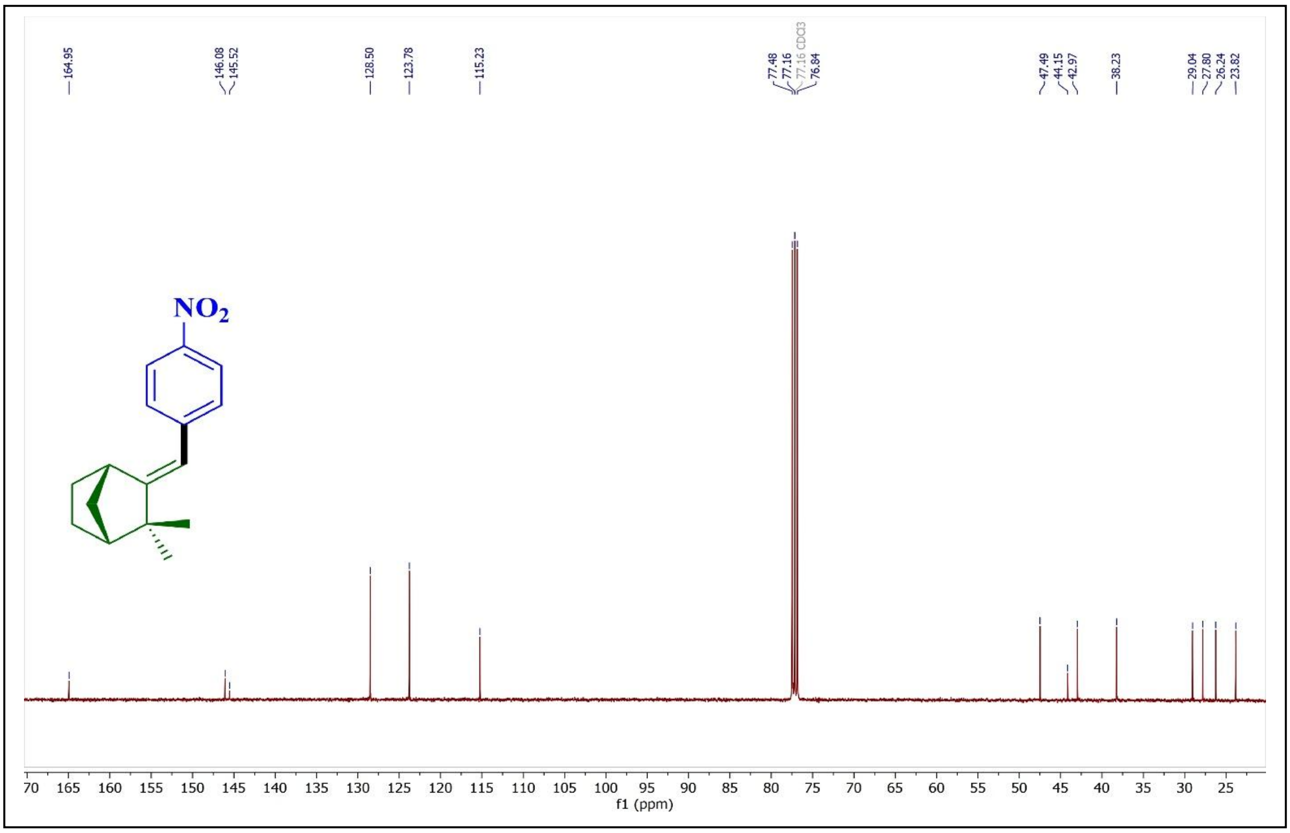

Figure S10. ^{13}C NMR spectrum (101 MHz, $CDCl_3$) of compound **3b**.

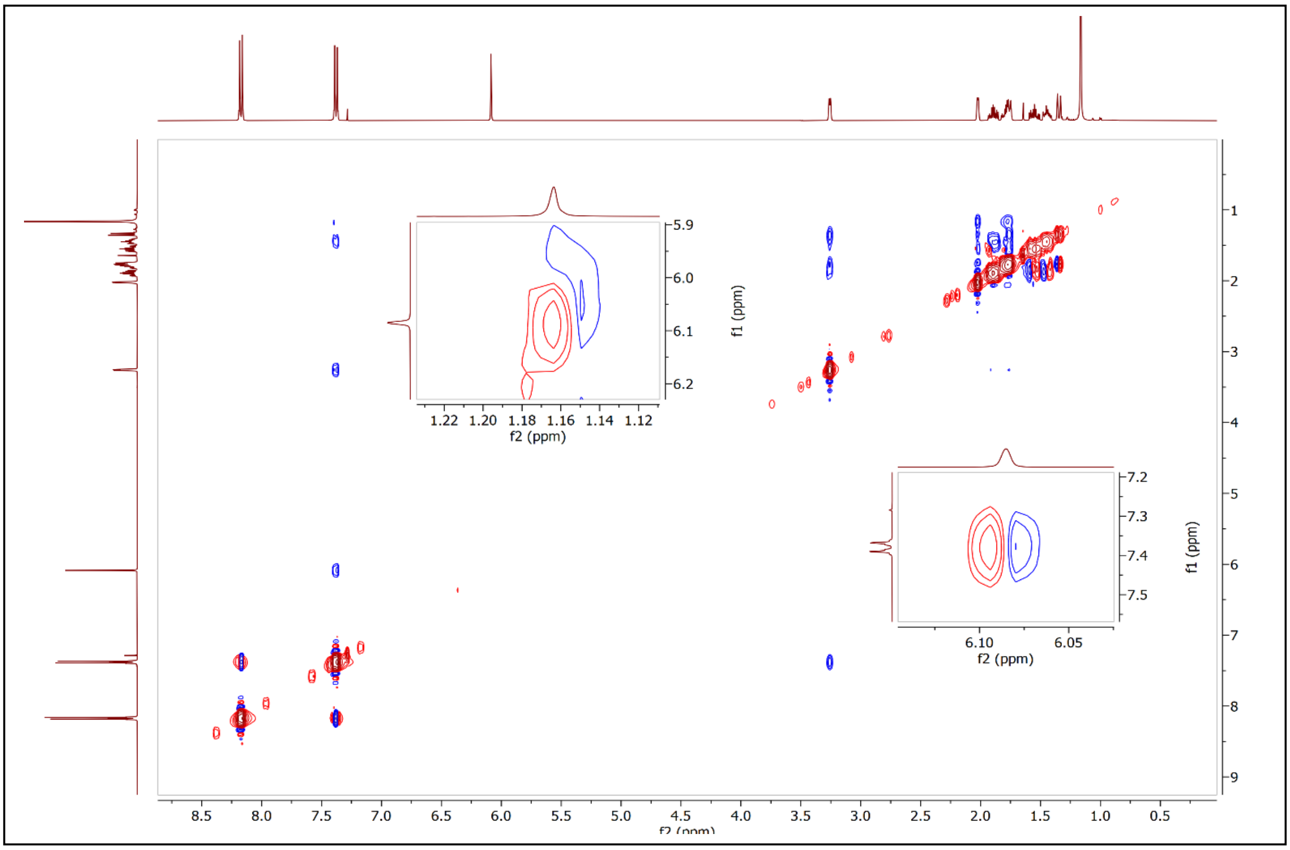

Figure S11. H,H-COSY NMR spectrum of compound **3b** in $CDCl_3$.

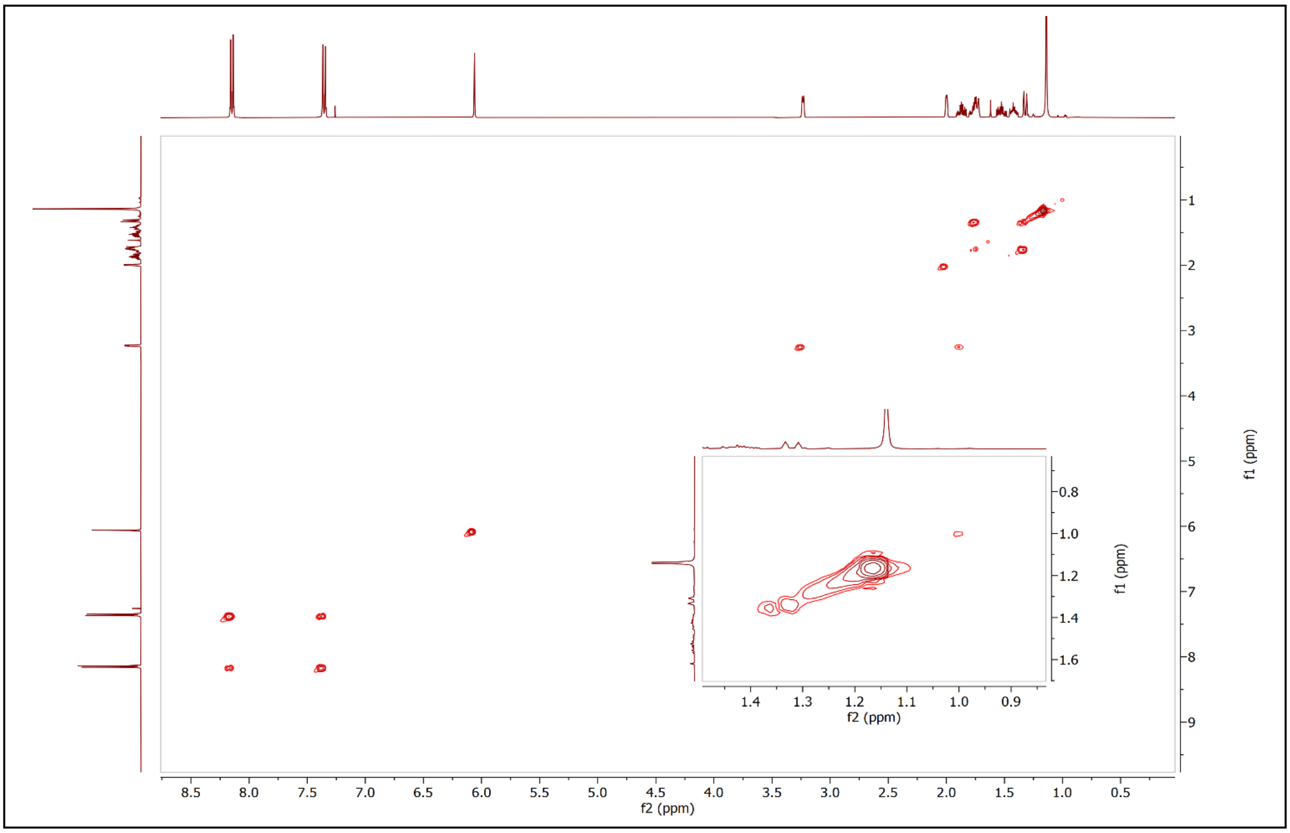

Figure S12. NOESY NMR spectrum of compound **3b** in $CDCl_3$.

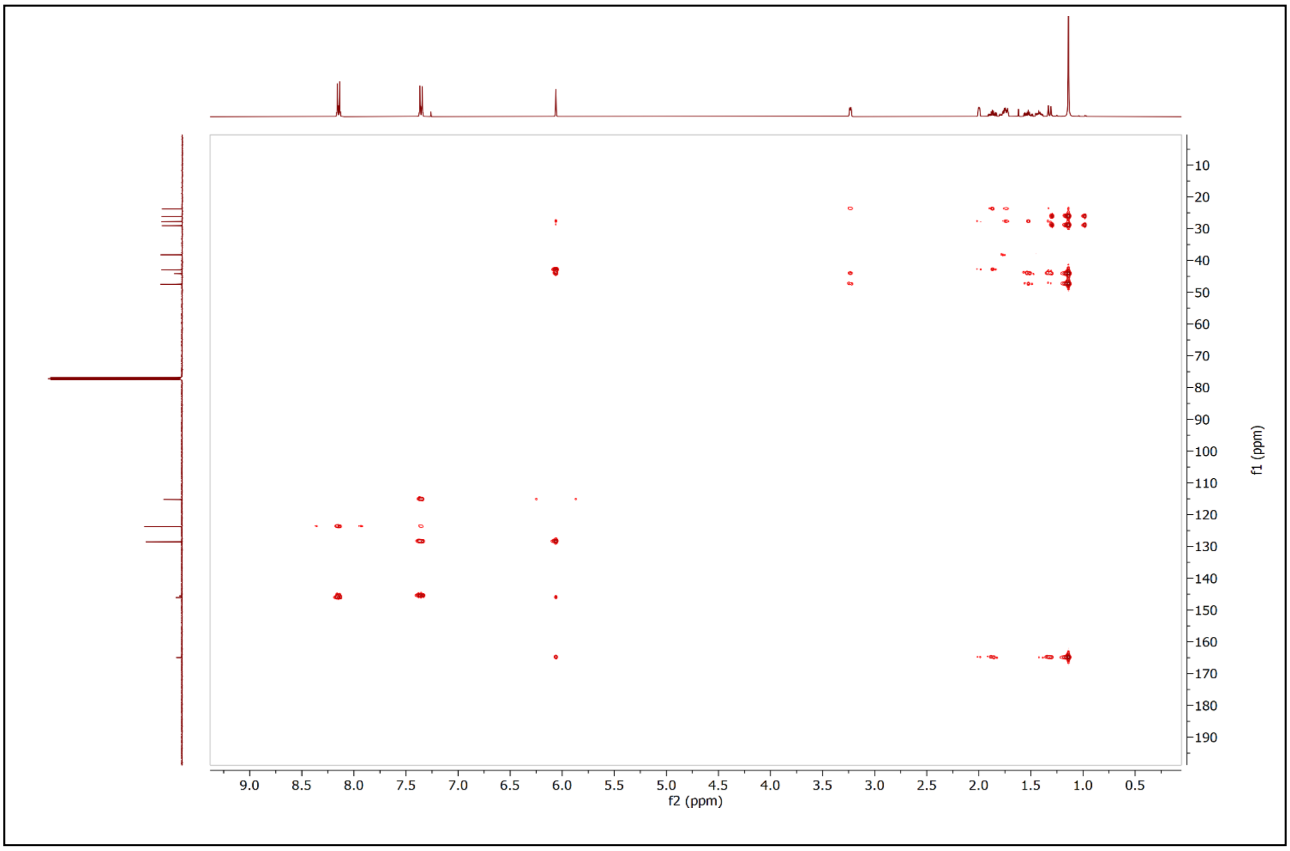

Figure S13. HMQC NMR spectrum of compound **3b** in $CDCl_3$.

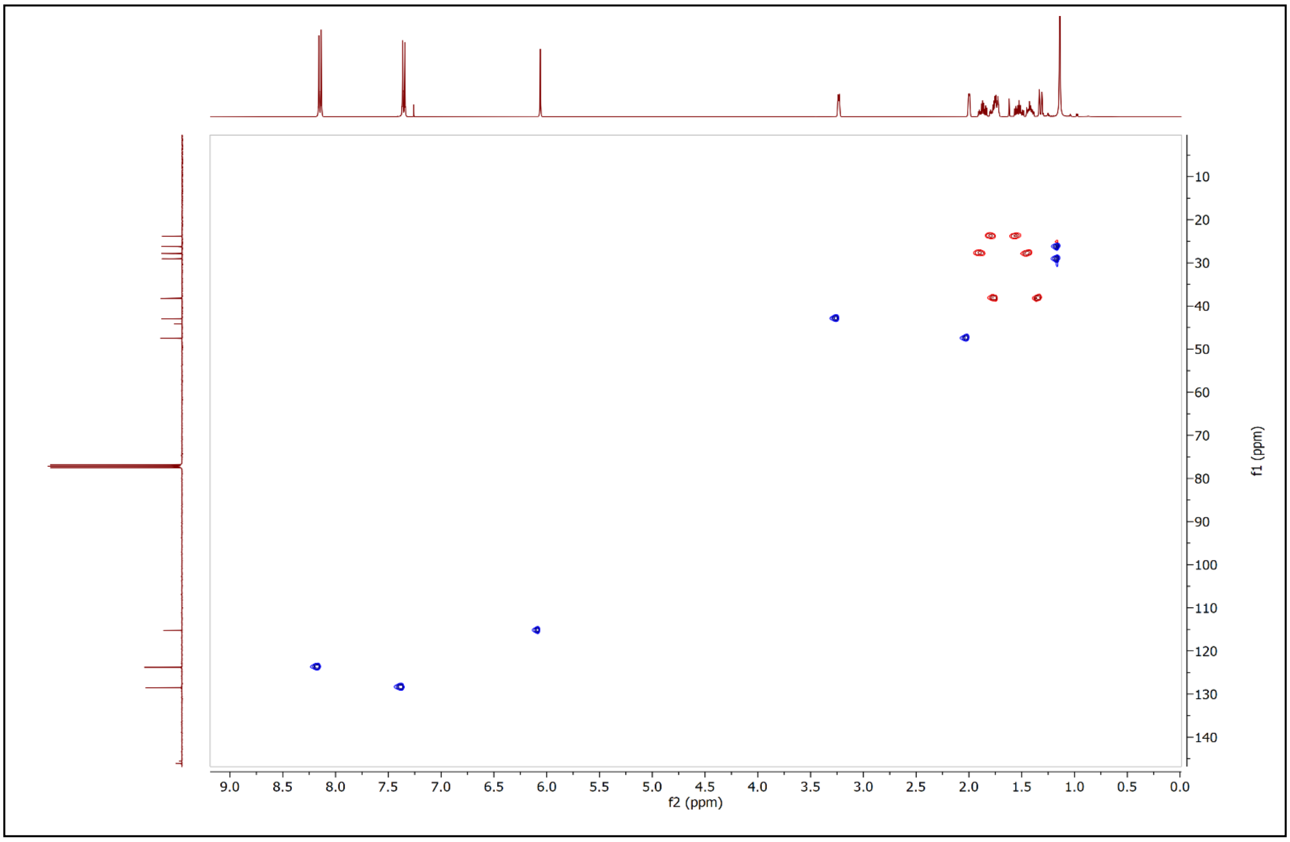

Figure S14. HMBC NMR spectrum of compound **3b** in $CDCl_3$.

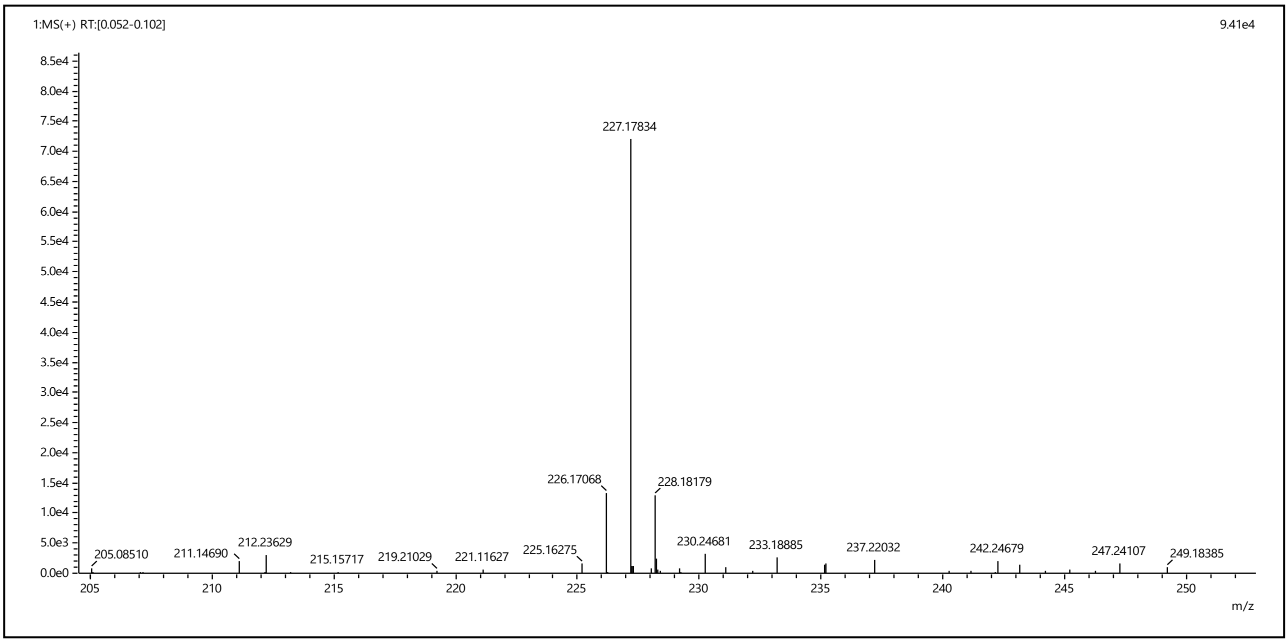

Figure S15. Mass spectrum of compound **3c**.

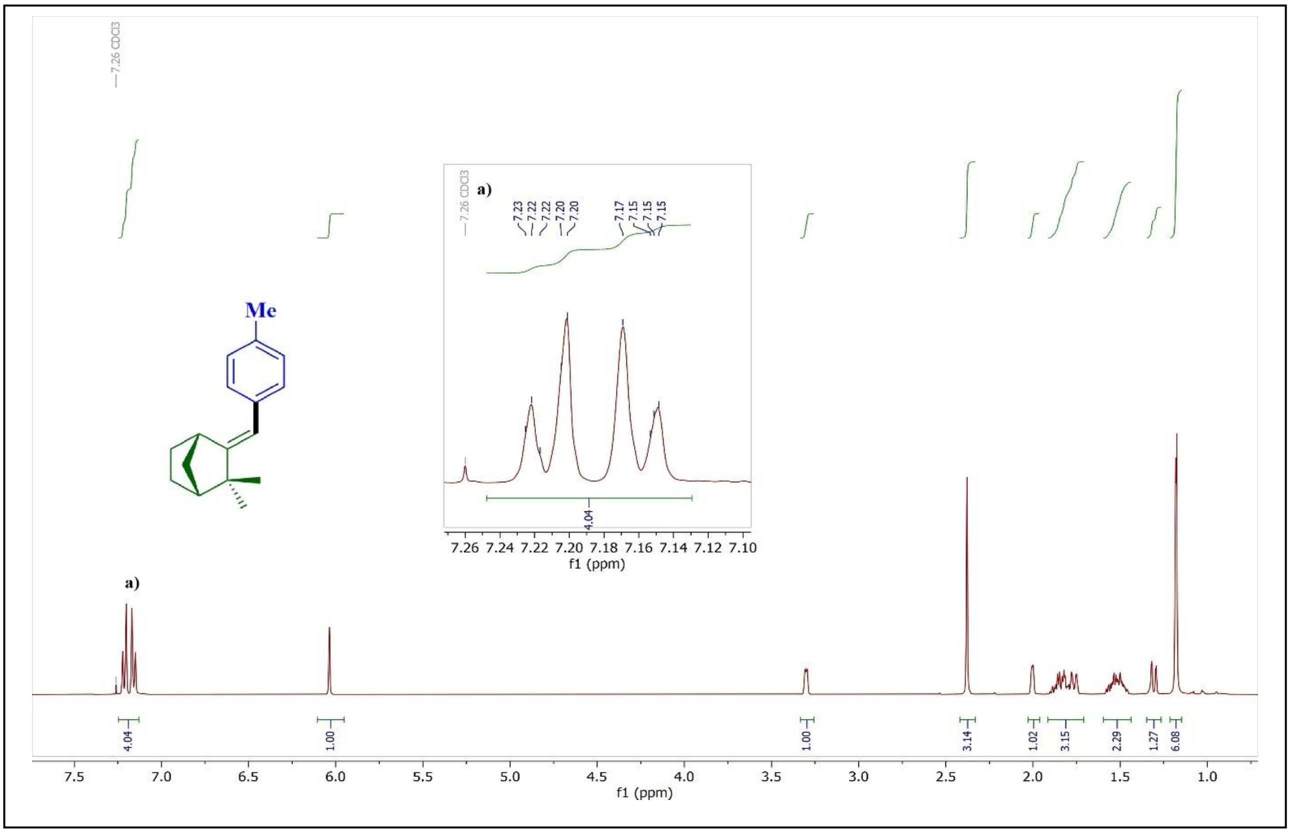

Figure S16. ^{1}H NMR spectrum (400 MHz, $CDCl_3$) of compound **3c**.

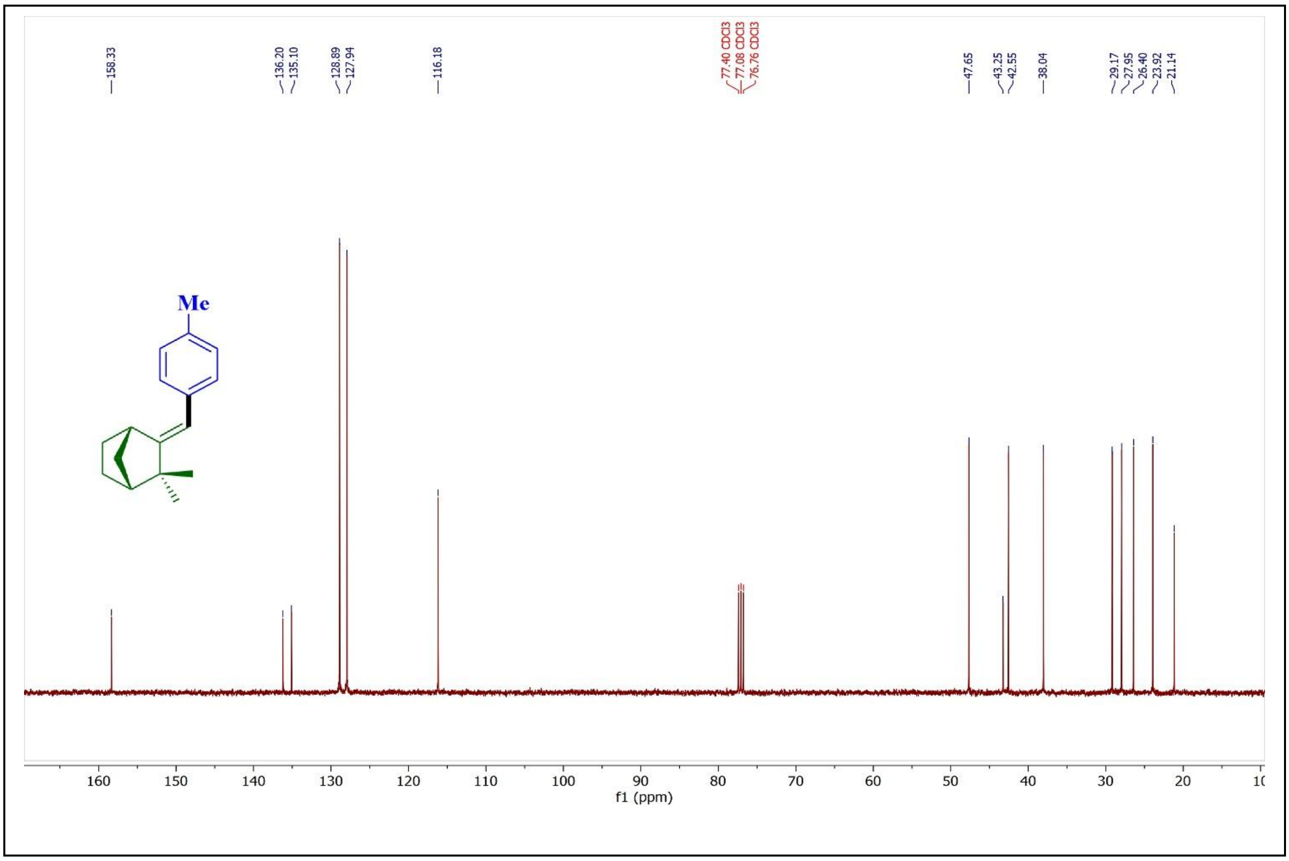

Figure S17. ^{13}C NMR spectrum (101 MHz, $CDCl_3$) of compound **3c**.

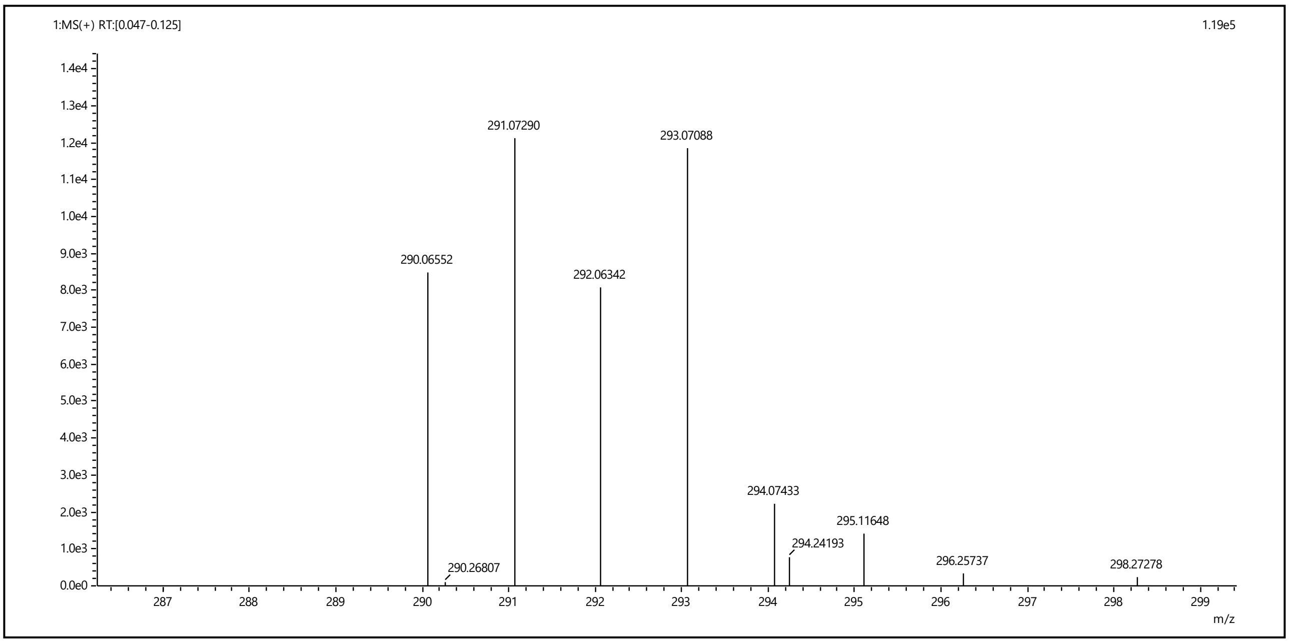

Figure S18. Mass spectrum of compound **3d**.

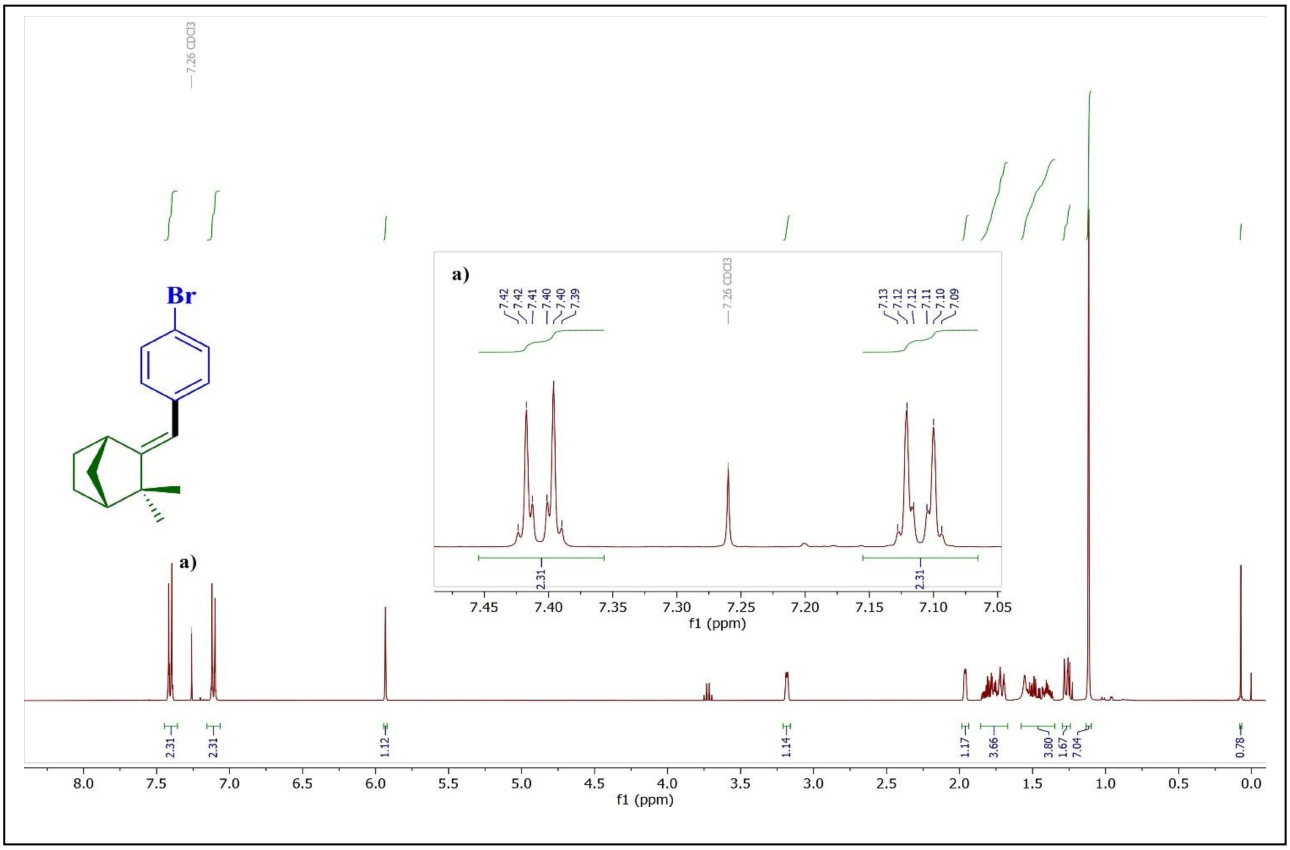

Figure S19. ^{1}H NMR spectrum (400 MHz, $CDCl_3$) of compound **3d**.

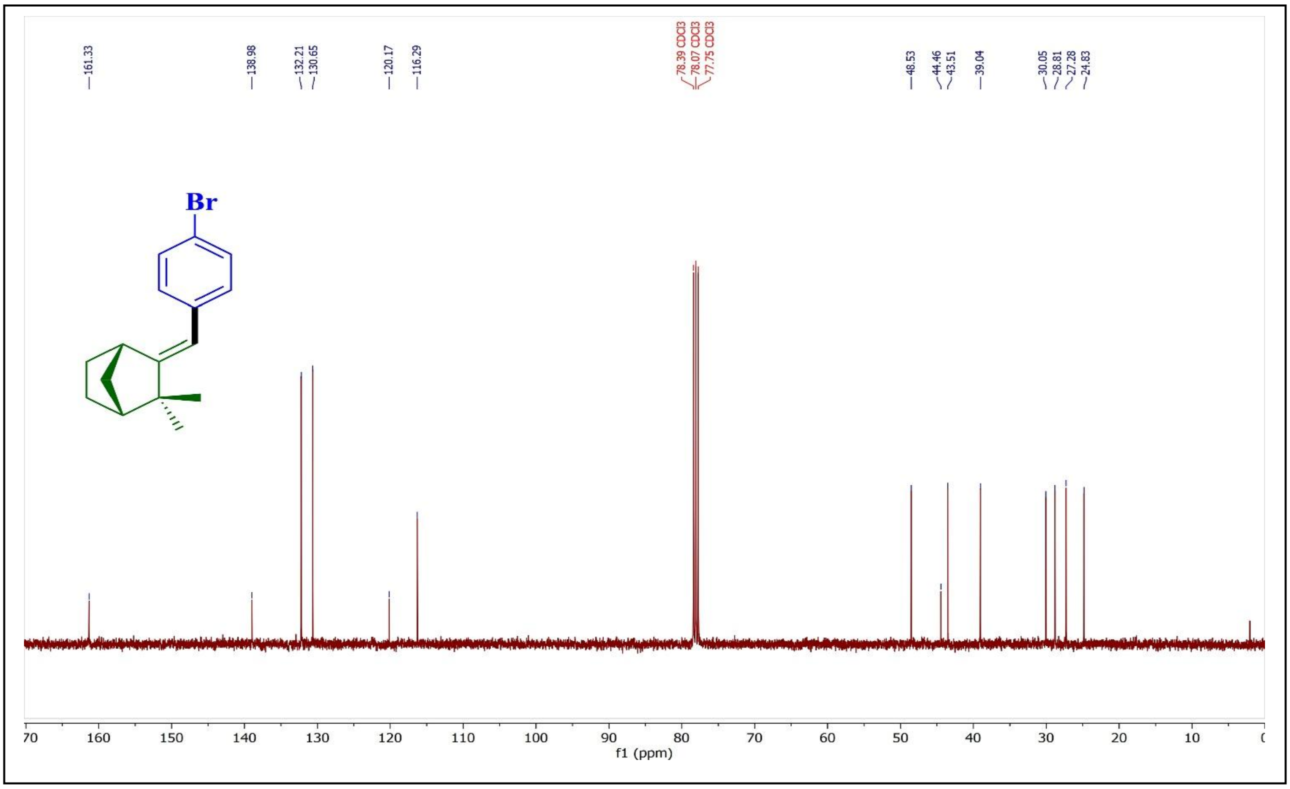

Figure S20. ^{13}C NMR spectrum (101 MHz, $CDCl_3$) of compound **3d**.

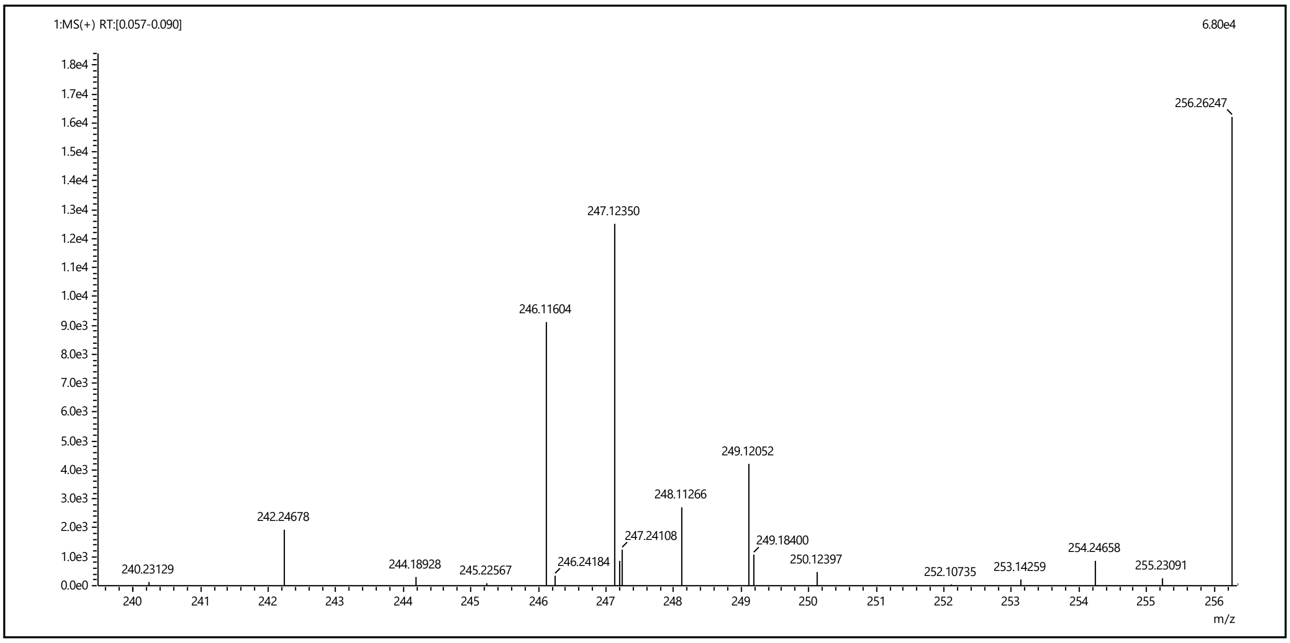

Figure S21. Mass spectrum of compound **3e**.

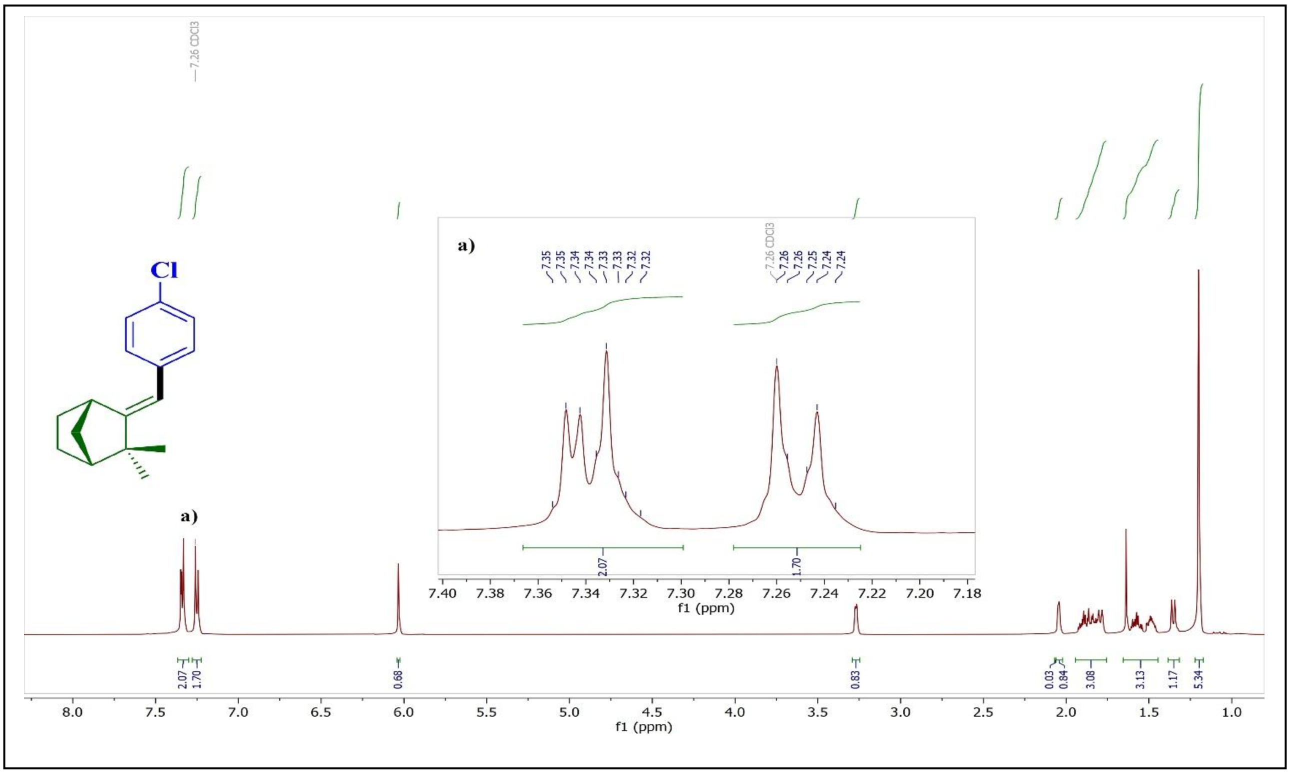

Figure S22. ^{1}H NMR spectrum (400 MHz, $CDCl_3$) of compound **3e**.

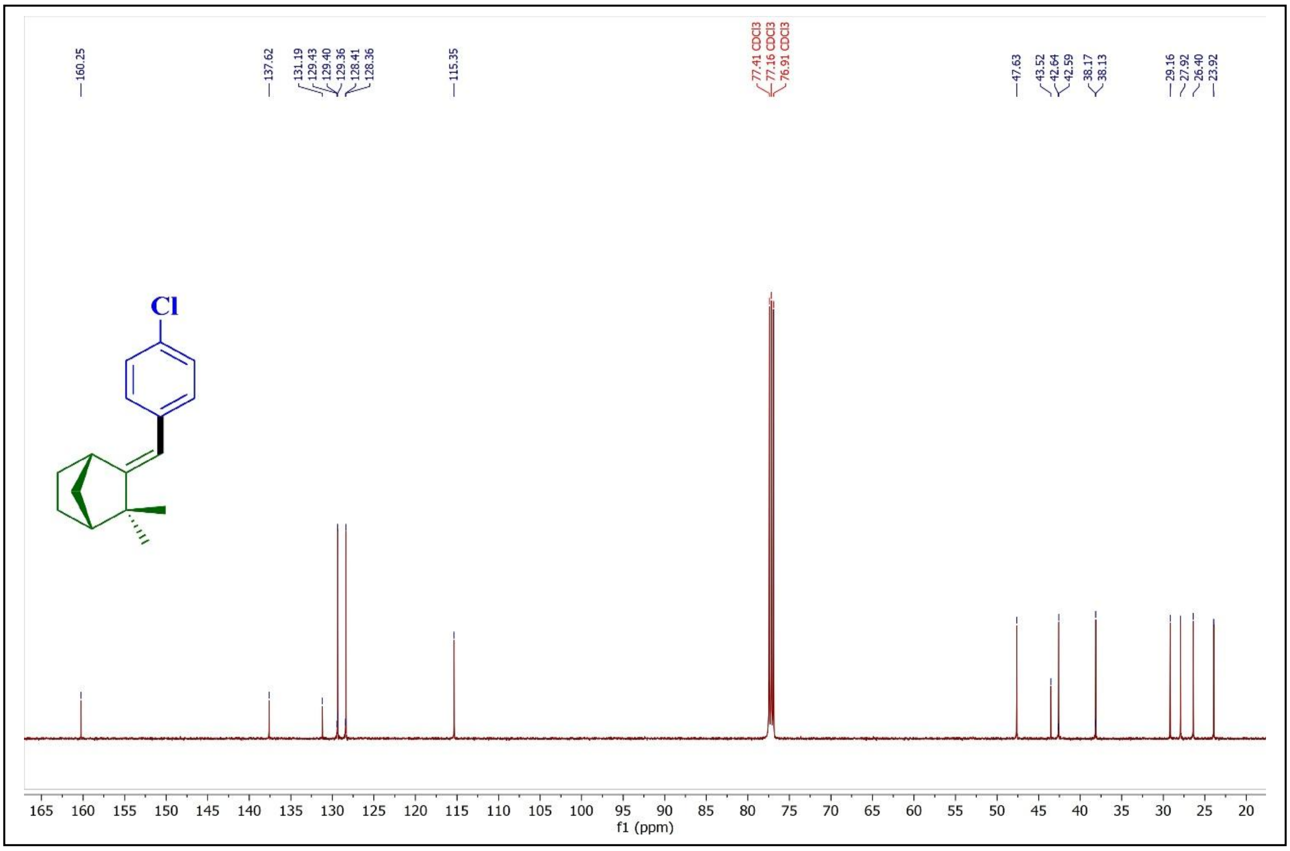

Figure S23. ^{13}C NMR spectrum (101 MHz, $CDCl_3$) of compound **3e.**

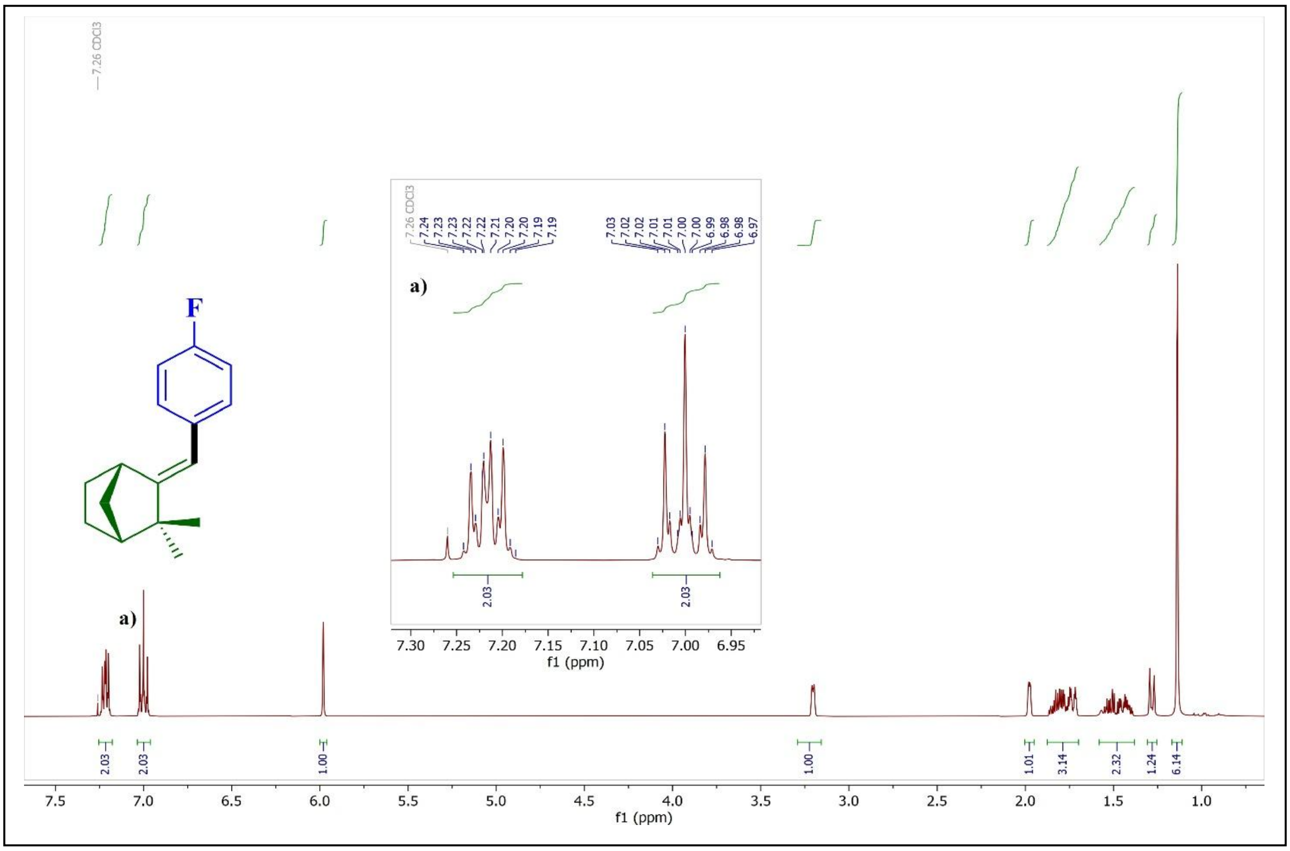

Figure S24. ^{1}H NMR spectrum (400 MHz, $CDCl_3$) of compound **3f**.

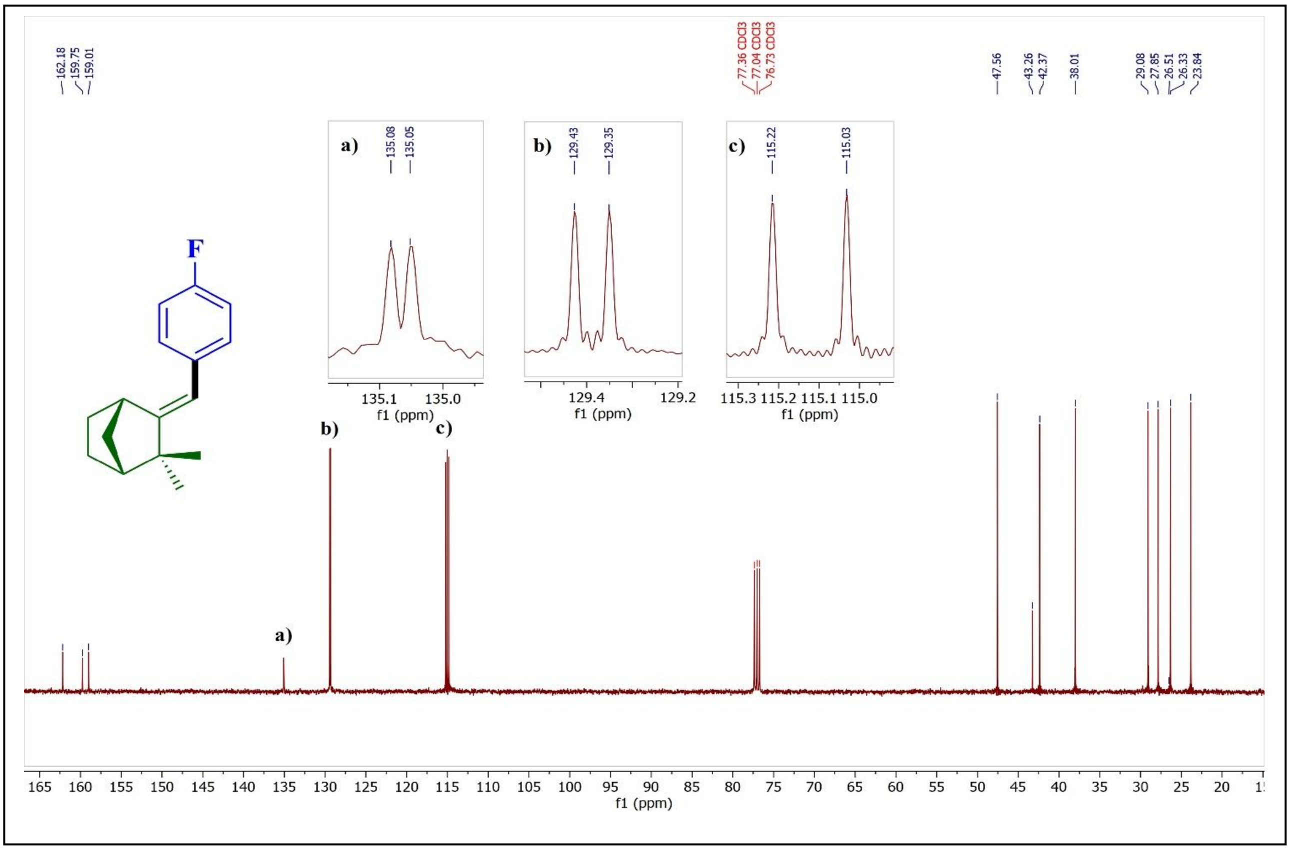

Figure S25. ^{13}C NMR spectrum (101 MHz, $CDCl_3$) of compound **3f**.

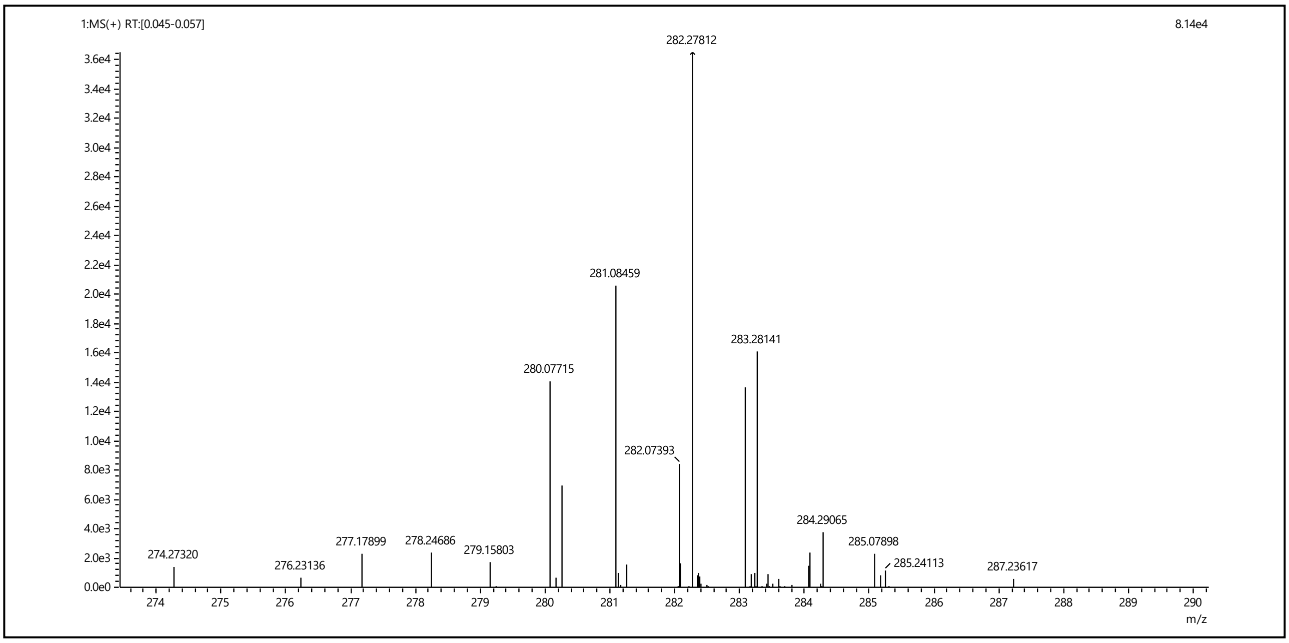

Figure S26. Mass spectrum of compound **3e**.

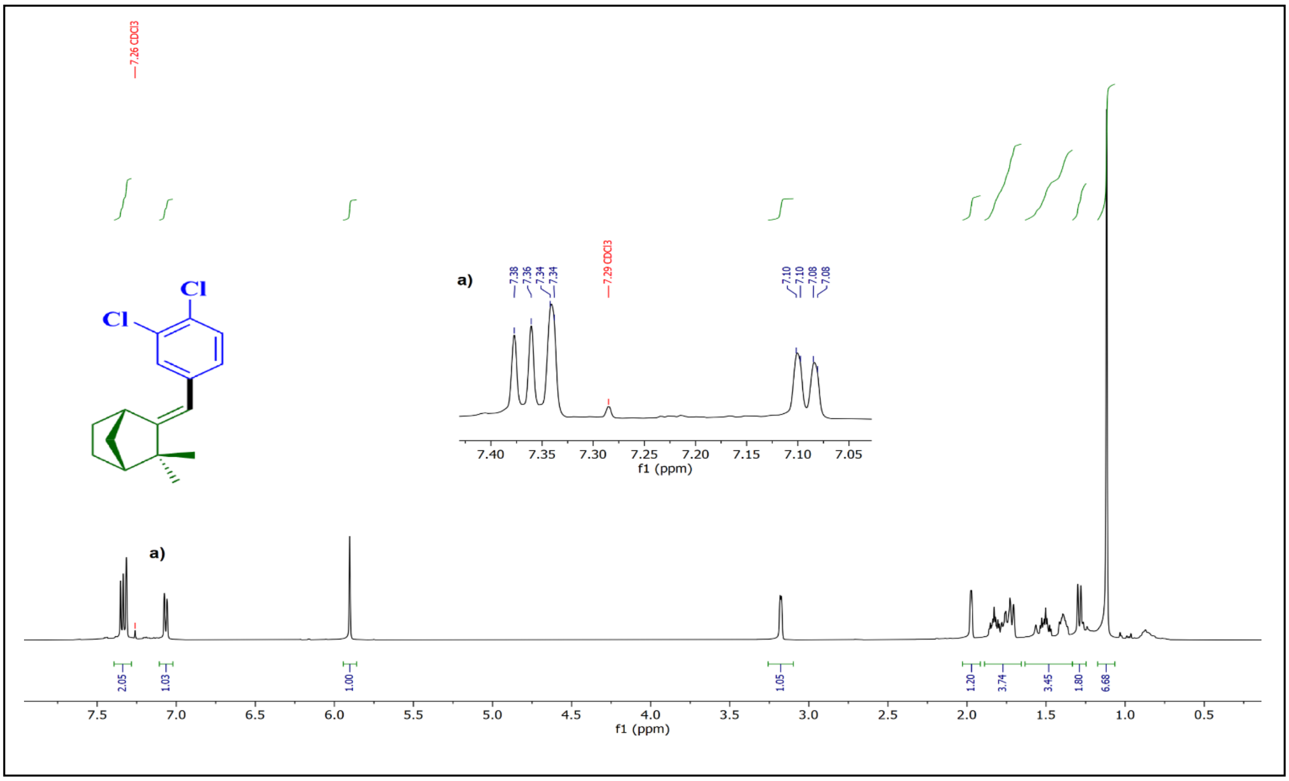

Figure S27. ^{1}H NMR spectrum (400 MHz, $CDCl_3$) of compound **3g**.

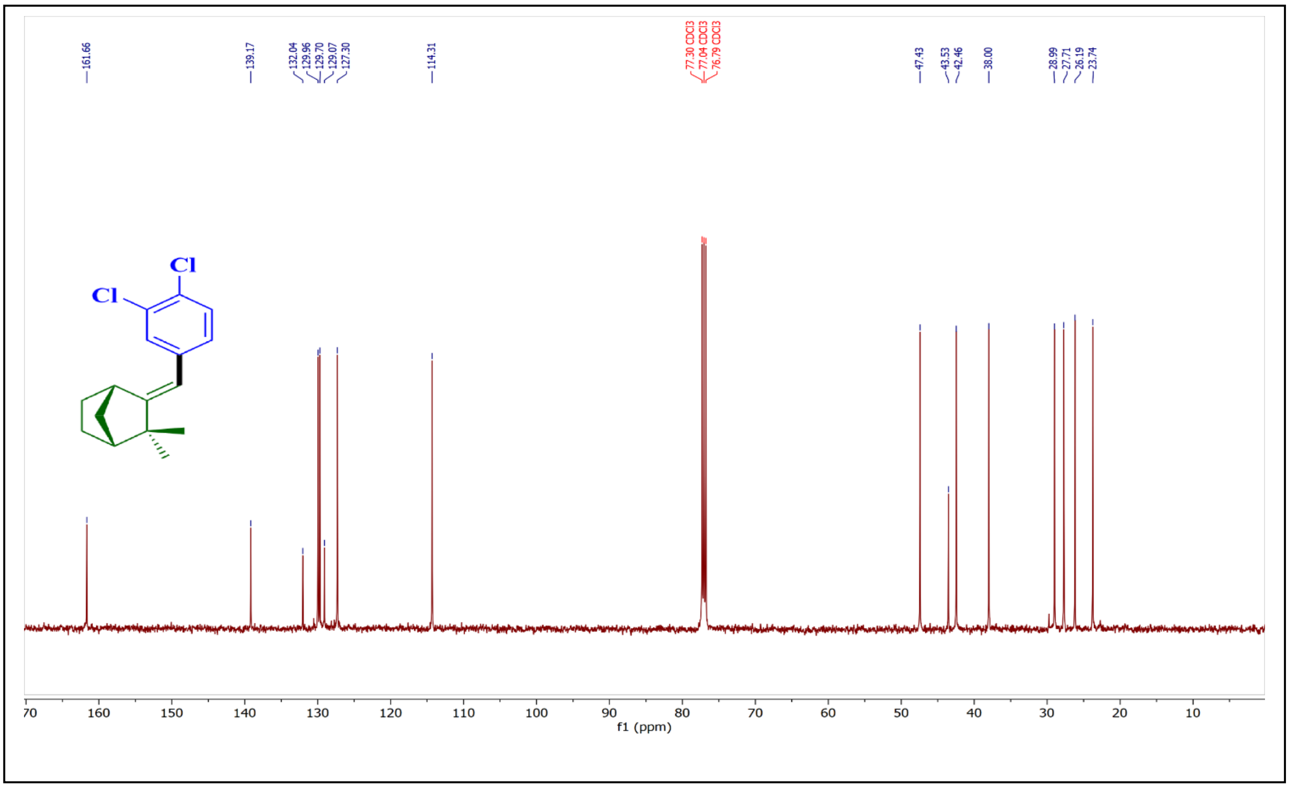

Figure S28. ^{13}C NMR spectrum (101 MHz, $CDCl_3$) of compound **3g**.

6. Supplementary References

[1] Still, W.C., Kahn, M. and Mitra, A. (1978) "Rapid chromatographic technique for preparative separations with moderate resolution," *The Journal of Organic Chemistry*, 43(14), pp. 2923–2925. Available at: https://doi.org/10.1021/jo00408a041.

[2] Angnes, R.A. *et al.* (2018) "Non-Covalent Substrate Directed Enantioselective Heck Desymmetrization of *cis* -Cyclohex-4-ene-1,2-diol: Synthesis of all *cis* Chiral 5-Aryl-cyclohex-3-ene-1,2-diols and Mechanistic Investigation," *Advanced Synthesis & Catalysis*, 360(19), pp. 3760–3767. Available at: https://doi.org/10.1002/adsc.201800785.

[3] Souza, E.L.S. de, Chorro, T.H.D. and Correia, C.R.D. (2023) "Thermal analysis of arenediazonium tetrafluoroborate salts: Stability and hazardous evaluation," *Process Safety and Environmental Protection*, 177, pp. 69–81. Available at: https://doi.org/10.1016/j.psep.2023.06.082.

[4] Yong, W. *et al.* (1991) "Arylation of Camphene with Arenediazonium Salts Catalyzed by Palladium Acetate," *Synthesis*, 1991(11), pp. 967-969. Available at: http://doi.org/10.1055/S-1991-26619
